# Global approaches to older abuse research in institutional care settings: A systematic review

**DOI:** 10.1371/journal.pone.0290482

**Published:** 2025-03-10

**Authors:** Maria Agaliotis, Tracey Morris, Ilan Katz, David Greenfield

**Affiliations:** 1 School of Health Sciences, Western Sydney University, Campbelltown Campus, New South Wales, Australia; 2 Masters of Public Health (University New South Wales), Sydney, Australia; 3 Social Policy Research Centre (SPCR), University of New South Wales, Sydney, Australia; 4 School of Population Health, Faculty of Medicine, University of New South Wales, Sydney, Australia; North South University, BANGLADESH

## Abstract

**Background:**

Over the last two decades, abuse of older adults in institutional settings has been underestimated due to challenges in defining and responding to the issue. This systematic review aims to analyze empirical studies on measuring abuse of older people residing in a long-term care facility, specifically staff-to-resident abuse.

**Methods:**

Following PRISMA guidelines, we searched 10 databases from January 2005 till June 2024. Inclusion criteria encompassed World Health Organization-defined abuse types (physical, psychological, financial, sexual and neglect, intentional or unintentional), reported by staff, residents, family, or public registries, with methodological critical assessment.

**Findings:**

In the last 18 years, 22 studies from eight counties examined of staff-to-resident abuse, with significant heterogeneity in definitions, reporting sources, and measurement tools. Quality of studies varied, lacking consistency. Relatives and staff typically report highest abuse rates, while residents report fewer incidents, even with fewer incidents of observed abuse. Registries tend to capture extreme cases, resulting in lower reported prevalence rates, particularly of physical or sexual abuse and neglect. Physical abuse was the most reported, with 81 different descriptors identified and varying recall periods. Staff witnessing abuse ranged from 44% over four weeks to as low as 1.4% over 12 months, posing challenges for data interpretation.

**Conclusion:**

These variations in study methodologies impacted the ability to synthesise the findings making it difficult to estimate a global prevalence rate of aged care abuse. From the analysis, we develop an Aged Care Abuse Research Checklist (ACARC) as a first step towards achieving a global standardized, evidence-based methodology for this field. Doing so will normalize processes within organizations and the community, allowing early interventions to change practices, reduce the risk of recurrence and improve resident quality of care and workplace cultures.

**Registration Number:**

PROSPERO CRD42018055484.

## Introduction

Older people have higher risks of isolation, fragility, impaired cognitive function, and lack of social support structures; individually, and collectively, these issues make them vulnerable to maltreatment or abuse, most often from persons in trusting relationships [1]. Maltreatment and abuse can contribute to long term physical and psychological harm including stress, injury, depression, and increased mortality [2].

Recent Organization for Economic Cooperation and Development (OECD) data estimates between 6 – 20% of people aged 80 and over currently reside in institutional settings, and by 2050 this is likely to double [3]. This change is in part driven by the fact that the global population of people aged 60 and over, will increase from 10% in 2022 to 16% by 2050 [4]. Institutional settings can range from independent living facilities, assisted living communities, nursing homes and continuing care retirement facilities. Abuse can be committed by staff-to-resident, resident-to-resident, or visitor-to-resident [5].

We do know rates of abuse are reported to be higher among the vulnerable dependent older adults living in institutional settings, compared to older people in the general community [5], and yet many instances go unreported. A 2019 systematic literature review found two in three residential unit staff self-reported committing abuse in the last year [5], while a recent review found healthcare workers were more likely to witness violence than perpetrated, with the highest levels of verbal abuse in nursing homes and neglect and financial abuse in home care [6]. Although evidence of extensive abuse of older adults is well established, challenges in defining, identifying, and responding to it restrict our ability to address the issue. In 2002, some clarity was brought to the problem by the World Health Organization (WHO), defining older adult abuse as ‘elder abuse’, and described it as an intentional or inappropriate act, single or repeated, causing distress or harm to an older adult [7]. Types of abuse include physical, psychological, or emotional, financial (or financial exploitation), sexual and neglect, intentional or unintentional [7]. Over the last ten years, there have been consistent calls to understand how to standardize, and measure rates of abuse among older adults [5,6,8–12].

We know that over the last two decades, due to varying definitions and social norms across the world, the rate of abuse among older adults in institutional settings has been underestimated [10,11,13,14]. In short, our understanding of the prevalence of abuse of older adults is significantly limited and recent descriptions of instruments used to examine staff-to-resident abuse in residential care settings need a more thorough standardized investigation since reporting abuse is an essential part of public health, and reports of abuse the responsibility of all members of the community [5,10,11]. Understanding the quality of abuse measurement tools among older adults [10,11] by undertaking a systematic review and examining all potential modes of reporting older age abuse (staff, resident, relatives, or community [via registries including whether allegations or sustained acts of abuse]) within long term aged care institutional settings will provide a clearer picture of the how to better standardize the methodological approaches to measuring older age abuse in institutional settings.

Overall, the study aimed to investigate and develop common standard research criteria to advance the methodological rigor and practical viability approaches when measuring older abuse within institutional settings. Four guiding questions direct the review: (1) what are the study characteristics? (2) what are the methods and measurement tools that have been used and are they valid and reliable? (3) what has been the impact of methodology on the results? and (4) what is the level of quality of these studies?

## Methods

### Search strategy

A systematic quantitative review protocol was developed according to the PRISMA [15] (S1 Checklist. PRISMA Checklist 2020) and registered (SYSTEMATIC REVIEW REGISTRATION NUMBER: PROSPERO registry number: CRD42018055484, https://www.crd.york.ac.uk/PROSPERO)[16] (S1 File. Prospero Registration Systematic Review Protocol). Ten academic databases (S2 File. Selected electronic databases) were searched. The keyword search was informed by Lindbloom et al. (2007) [14] and a Cochrane review by Baker et al.(2016) [17] (S3 File. Search terms and strategy). In addition to this search, full paper copies of potentially relevant articles were retrieved, and their reference lists were screened.

### Eligibility criteria

The inclusion criteria included: observational studies reporting any incidence or prevalence data on any type of abuse as defined by the WHO (2022) [physical, psychological, financial, sexual abuse and neglect]; as observed or committed abuse on older participants residing in long term institutional care facilities including assisted, independent or extended living facilities or care units, and residential or a nursing home; staff-to-resident abuse from ‘health care professional’ or ‘staff member’ to ‘patient’ or ‘resident’. Research articles were limited to full-text English language and published from January 2005 till May 2020. This timing coincides with the last systematic review on abuse among older residents residing in nursing homes conducted by Lindbloom et al (2007). Additional searches were conducted using the same academic databases to retrieve studies published between May 2020 to June 2024. We also excluded studies based on study design such as single case reports; case series; and discussion or opinion pieces (S1 Table).

### Data extraction and data analysis

Research results were merged and organised using reference manager software, Endnote (X20; Clarivate, Philadelphia, PA, USA) and Microsoft Excel 2020 (Microsoft Corp., Redmond, WA, USA). Duplicate articles were identified and excluded using Endnote X20. Titles and abstracts obtained from the search were screened by two reviewers (MA and TM) using Microsoft Excel 2020. Data were extracted by one reviewer (TM) and independently audited by a second (MA). The data extraction was guided by an analytical framework using the elements of epidemiological methodology used in prevalence studies [18] (S4 File). The framework characteristics and elements form the header columns for presented tables (Table 1) and rows form the information extracted from each article. Disagreement or ambiguities were resolved by consensus. Descriptive tables were developed based on the study recruitment methodology, that is who reported the abuse (staff, residents, relatives, or community) (S5 File). The subheading columns were structured based on the examining, study characteristics, methodology characteristics and results (S5 File).

**Table 1:** Summary of study characteristics via staff, residents, relatives and community reporting abuse.

STUDY DETAILS	DESIGN		SAMPLE				METHODOLOGY				QUALITY
**Author, Year, Country, [Author Extracted and Date]**	**Types of Abuse**	**Study Design**	**Recruitment, Definition NHs, N (%), Study Population N (%), Response Rate (RR) %**	**Definition, Age mean (±sd), [Range](%, F)**	**Yrs. Resident, Professional, Experience mean (±sd) [Range]**	**Dependency N (%)**	**Method of collecting data, Distributed, No: of items Source, Questionnaire**	**Experienced (E)/Observed (O)**	**Recall Months**	**Frequency of Abuse**	**Validity of Tool**
**Staff**											
1. Ben Natan M et al. 2010, IL [26] [MA & TM 14 April 2017]	Overall, P, Psych, F (exploitation), S N	CS	Rand LTF LTF, 300, (8%), S:600 (NR%), RR: 85%, Facility Directors: 24, RR: 91.6%	R: Elderly Patients, NR, [NR], (F: NR) S: Staff members (incl. nurses, nursing aids, administration & facility directors), NR, [20–65]	R: NR, NR, [NR]	R: NR	Q, Researcher, SQ: Items: 5 parts	S: D	12	S: Number: 1 -5, 6-10, 11 -16, 17 or more	NR
			40 – 49, (F:81.6%)	Overall (Yrs.) S: 13.8 (NR)		Part 2: Reporting incidents of violence					
					[NR]		Daly & Jogerst (2005), Iowa Dependent Adult Abuse Nursing Home Questionnaire				
					Present NH, ≤ 5 61.4%						
2. Blumenfeld Arens OB et al., 2017, SW [47] [MA 10 March 2020]	P, Psych (Emotional), N	CS	Rand NH: NHs (SCU, Non-SCU or other): 1,600. S: 6,000, (76.7%), RR: NR%	R: Residents, 84.6 (±3.0), [NR], (F:9.8%). S: Direct care workers, 43.1, (±12.3), [NR], (F:NR)	R: NR & S: NR	R: NR	Q, NR, SQ Items: 42 Malmedal et al. (2009)	S: O	4 Wks.	S: How often: Never to more	Cronbach’s alpha 0.78, Face-to-face content
										than once a week (4-point Likert scale)	Content validity
											Internal validity
											External validity
3. Botngård A et al. 2020, NO [48] [MA 10 March 2020]	Overall, P, Psych, F (Material), S, N	CS	Rand NH NHs (CRE) 939 [7.8%]	R: Residents NR [NR], (F: NR) S: Nursing staff, NR [16–73], > 31-75, 71.1%, (F: 91.5%)	R: NR & S: NR	R: NR	Survey, Coordinator distributed SC from SQ: Items 35 Castle (2012) modified, Verbal: Clarke & Pierson (1999), Psych: Lachs & Pillemer (2004), Caregiver: Federal Register (1991), Medication: Chambers (1999), Material exploitation: Rabiner et al. (2006), Sexual: The National Center on Elder Abuse (1998)	S: D & O	12	S: How often Never to Repeatedly	Generalisability
		S: 6337, (58.3%), RR: 60.1%								(3-point Likert-type scale)	Validity tested
											Internal validity
4. Buzgová, R., & Ivanová, K. 2011 CR [48] [MA & TM 14 April 2017]	Overall, P, Psych, S, N (Care)	CS, (interviews and questionnaires)	Rand NH, Senior Homes 24 (50%), NR: RR%, R: 488/3597, (27% selected), RR: NR%	R: clients, NR (±NR)	R: Length of stay, NR [<4 - > 10], R: < 4, 62.1%, S: Present NH, NR, [<4 - > 8], > 8 years, 173, 38.1%	R: Self-sufficiency	R: I, NR, SC from SQ: 32 questions (26 listed forms of elder abuse), Buzgová & Ivanová et al. (2009), WHO (2002)	R: D & O & S: D & O	R & S: 12	R: How often (number)	Validity tested
		S: 477/ 1446, (NR%), RR: 64%	[60 - > 75], (F: 74.8%)		(Barthel ADL Index)	S: Q, Anonymous, NR, SC from SQ, 40, Buzgová & Ivanová et al. (2009) & WHO (2002)				S: Never, Once, repeatedly (3-point Likert-type scale)	
				>75 years, 350 (71.7%)		262, 53.7%					
				S: DCE, NR (±NR)							
				[18 to > 35], (F: 96.9%) > 35 years, 333 (73.4%)							
5. Castle N. 2012, USA [37] [MA & TM 14 April 2017]	P, Psych (& verbal), F (material exploitation), S, Other: Caregiver abuse Medication abuse	CS	Rand Prof: Registry NHs, NR	R: Residents NR, [NR], (F: NR) S: Nurse aides or Certified Nursing Assistants (CNAs) 32.5 (±8.1) [NR], (F: 91%)	R: NR S: Present NH S: 1.1 (±3.1)	R: NR	Q: Mail out, NR	S: O	3	S: Number. Never (0), Once (1) or more (number)	Face validity
		125.1 (±72.2) beds. S: 7,000, (49%), RR: 64%				SC from SQ: Items 46 (28 measured abuse), Verbal: Clarke & Pierson (1999)					Content validity
							Psych: Lachs & Pillemer (2004) Caregiver: Federal Register (1991) Medication: Chambers (1999) Material exploitation: Rabiner et al. (2006), Sexual: The National Center on Elder Abuse (1998)				
6. Castle N & Beach S. 2013, USA [38] [MA & TM 14 April 2017]	P, Psych (verbal), F (Material exploitation), S, O: Caregiver abuse, O: Medication abuse	CS	Rand Prof: Registry Als, 1470 (nr%) RR: NR, S: 895 (NR%), RR: 63%	R: Residents, NR (±NR), [NR] (F: NR), S: Nurse aides, 31.4 (±8.1) [NR], (F: 94%)	R: NR, NR, [NR]	R: NR	Q: Mail out, NR SC from SQ, 46 (28 measured abuse), Verbal: Clarke & Pierson (1999), Psych: Lachs & Pillemer (2004), Caregiver: Federal Register (1991), Medication: Chambers (1999)	S: O	3	NR	Face validity
				S: NR, NR, [NR]		Material exploitation: Rabiner et al. (2006), Sexual: The National Center on Elder Abuse (1998)					Content validity
7. Gil AP & Capelas ML, 2022, PT [51] [MA 21 June 2024]	Overall, P, Psych, F, S, N, Other: Global	CS (interviews)	One council in the metropolitan area. Care homes. F: 16 (50%) RR: NR%. S: 186 (NR %), RR: 88.4%	R: Residents, NR (±NR), [NR], F: (F: NR%)	R: NR, NR [NR]	R: NR	NR, Q: NR (self-completed), SC from SC, 31	S: D & O	12	Ever	NR
			S: Care workers, 20 (51.1%), 47 (±NR), [21 - 68] (F: 94.0%)	S: 10 years (NR) [NR] Permanent contract		Rabold and Goergen (2013) and Drennan et al. (2012), NR for types of abuse	(excluded including others including S)				
8. Malmedal W et al, 2009, NO [53] [MA & TM 14 April 2017]	Overall, P, Psych (Emotional), F, N	CS	Rand NH, Nursing Homes, 51	R: Resident, NR	R: NR, NR, [NR]	R: Confused	Q, Staff. SC from SQ: Items: 42	S: D & O	4 Wks.	S: How often	Cronbach’s alpha; physical 0.57. Validity tested
		(31%). S: 780, (NR) RR: 78.9%	[NR], (F: 70%)	S: Overall (Yrs.)	59%	20 items on acts of inadequate care				Never to more than once a week	
				S: Staff members 40 (±13), [16-74] (F: 97%)	14 (NR) [0-45]		Based on several clinical research studies			(4-point Likert-type scale)	
					Present NH: 8 (NR)		Saveman BI et al. (1999), Goergen (2001) & Pillemer & Bachman-Prehn (1991)				
9. McCool JJ et al. 2009, USA [43] [MA & TM 14 April 2017]	Overall, P, Psych (Emotional), F, N	CS, (interviews and survey)	Nursing Facilities (2)	R: Resident, NR (±NR), [NR] (F: NR%), S: Staff members, NR (±NR), [18 - 71] (F: 86.5%) Nursing & administrative staff 29 (59.2%) Other staff 20 (40.8%)	R: NR, NR, [NR]	R: NR	Q, Staff, Postal, SC from SQ, 28	S: O	Ever	Ever in the current facility	NR
		Nursing Facilities		S: NR, NR (NR), [NR]			5 person experiences with suspected adult abuse and reporting, Clark-Daniels CL et al, 1990 & Oswald RA, 2004				
			2 (15%), RR: NR%S: 335, NR%								
			RR: 15%								
10. Moore S. 2016, UK [32] [MA & TM 14 April 2017]	P, Psych, F, S, N, Other	CS (interviews and survey)	5 new care homes for older people	R: Residents, NR (±NR), [NR], (F: NR%), S: Care staff, NR (±NR) [NR], (F: NR%)	R: NR, NR, [NR]	R: NR	Q: Mail out, Managers contacted Researchers	S: D	12	Never and ever	NR
		Local authorities with adult social services Private sector care and NHs 152 (22%), RR: NR%, S: 134, 189 (NR%), RR: 70.9% (average)		S: NR, NR, [NR]			SC, NR Department of Health, 2000				
11. Moore S. 2020, UK [33] [MA 21 June 2024]	Overall	CS (interviews and survey)	11 newly open NHs 11 (NR%), RR: NR%, S: NR, 429 (NR%), RR: 82.9%	R: NR, NR (±NR) [NR] (F: NR%). S: Care Staff, NR (±NR), [NR], (F: NR%)	R: NR, NR, [NR]	R: NR	Q: Mail out, Managers contacted, Researchers, SC, NR, Department of Health, 2000	S: O	12	Happened once, or repeatedly	NR
				S: NR, NR, [NR]						During day or night 12 months ago, 1 -3 years ago, More than 3 years ago	
12.Neuberg M et al. 2017, HRV [54] [MA & TM 14 April 2017]	P, Psych, F, S	CS	2 state and 2 private NHs & 2 Ext care units, NHs & Ext care units, NR (NR%)	R: Elderly Individuals	R: NR, NR, [NR]	R: NR	Q: NR, NR, SQ, 25, Drennan J, Lafferty A, Treacy MP, Fealy G, Phelan A, Lyons I, Hall P. Older People in Residential Care Settings: Results from a National Survey of Staff-Resident Interactions and Conflicts. NCPOP: University College Dublin, 2012	S: O	12	Never	Internal validity
		RR: NR%. S: Nursing Professionals: 200 (85.5%), RR: 85.5%	NR (±NR), [NR]. (F: NR%)	S: 20.0 (NR), [8.0-30.0]						Once	
				S: Nursing Professionals Qualifications						2 to 10 times	
				Bachelor: 39 (22.8%),						More than 10 times	
				Masters: 3 (1.8%)							
				Secondary: 129 (75.4%), 41.0 (±NR), [30.0-51.0], (F: 86.5%)							
13. Smith DE et al.2022, AUS [46] [MA 21 June 2024]	S	R CS	Sample of residential aged care services nurses enrolled to complete an e-learning course	R: Residents, NR (±NR), [NR], (F: NR%). S: EN & RNs (aged care nurses)	R: NR, NR, [NR]	R: NR	S: Online, nurses registered to course, SC, 7, SC survey instrument	S: D & O	12	Ever	NR
		Residential aged care services	Senior Management 20 (51.1%), NR (±NR), [35 – 64], (F: 91.1%)	S: NR (NR), [NR]							
			NR (NR%)		10 years’ experience: 26 (57.8%)						
			RR: NR%. S: EN & RN’s (aged care nurses), 167 (77.2%)								
			RR: 34.9%								
**Residents**											
1. Buzgová, R., & Ivanová, K., 2011, CR [49] [MA & TM 14 April 2017]	Overall, P, Psych, S, N (Care)	CS (interviews and questionnaires)	Rand NH, Senior Homes: 24 NHs	R: clients, NR	R: Length of stay	R: Self-sufficiency (Barthel ADL Index) 262 (53.7%)	R: I, NR, SC from SQ:	R: D & O & S: D & O	12	R: How often, (number), S: Never, Once, Repeatedly, (3-point Likert-type scale)	Validity tested
		(50%), R: 488/3597	[60 - > 75], (F: 74.8%)	NR, [<4 - > 10], R: < 4, (62.1%)			32 questions, 26 listed forms of elder abuse. S: Q, Anonymous, Items: 40				
			(27% selected)	>75, 350 (71.7%). S: DCE, NR, [16 to > 33]	S: Present NH		Buzgová & Ivanová et al.(2009) & WHO (2002)				
			RR: NR, S: 477/1446	(F: 96.9%), > 35, 333	NR, [<4 - > 8]						
			(NR%), RR: 64%	-73.40%	>8, 173 (38.1%)						
2. Cohen M et al. 2010, IL [44] [MA & TM 14 April 2017]	P [signs of abuse], Psych, S, N [signs of abuse & neglect of basic needs], Other: Signs of abuse: Exploitation & Disrespectful attitudes	CS (interviews and observation of health profiles and a list of maltreatment or abusive acts)	Hospitalised R	R: Hospitalised inpatients: 81.6 (±7.5), [70 - 99], (F: 64.8%). S: Staff: NR, [NR], (F: NR)	R: NR, NR, [NR]	R: ADLs: Totally dependent	I: (F-to-F), Social workers. SC from SQ: Items: 24, Listed maltreatment/abusive acts: Kottwitz & Bowling (2003)	R: D	12	R: ‘Maltreatment and abusive acts	Internality reliability
		Elderly homes, sheltered homes & NHs or ‘nursing (long-stay) departments’ of hospitals sheltered-home facilities		S: NR, NR &[NR]	15, (61.9%)	Wan, Tseng and Chen (2007) O: (Physical), Nurses Signs of Abuse Inventory and the Expanded Indicators of Abuse Questionnaire (Cohen, 2006, 2007)				‘Never’ to ‘almost all the time’.	Criterion validity
			NR, R: 71, (64.8%), RR: NR							Score: 0 to 72, R: Signs of Abuse Inventory ‘0’, ‘not at all’, to ‘4’, ‘extreme’ (4-point Likert-type scale)	
3. Habjanič A & Lahe D, 2012, SI [52] [MA & TM 14 April 2017]	P, Psych (mental), F	CS	Rand NH, Nursing Home: 7/10 NHs [Pool 28] R: 1,541 (41.0%) Randomly selected 200, RR: 81.5%. From NHs 42.7% (vs. community-based setting residents)	R: Nursing home residents, NR, [ ≥ 53], (F:82.8%)	R: NR	R: ADLs	I: (F-to-F), Researchers	R: D	6	R: Reported (Number)	Reliability tested & Validity tested
				75-84 years, 47.7%	S: NR	Occasional - Always	Nursing staff informed residents of study				
				S: Nursing home staff, NR, [NR], (F: NR)		104 (81.3%)	SC from SQ: Items: NR. Develop from examples primarily from:				
							The National Center on Elder Abuse (1998, Isola et al. (2008), Garre-Olmo et al. (2009) & Malmedal et al. (2009)				
**Relatives**											
1. Griffore RJ et al. [MA & TM 14 April 2017]	P, Psych, F, S, N, Other: Caretaker abuse	CS	Rand RDD Community RT	R: Age 65 or older resided in a facility that they defined as a nursing home	R: NR	R: NR	I: RDD, [Telephone Interview], SQ: Items: NR	RT: O	12	RT: Number None,1 or 2, 3 to 5, 6 to 10, More than 10	NR
			Nursing Homes or	NR, [≥53], (F: NR)	S: NR & RT: NA		Michigan Survey of Households with Family Members Receiving Long-Term Care Services (MLTCS)				
			Long-Term Care Services, NR, RT: 1002, (45.1%), (RR: NR)	S: Staff & Caregiver							
				NR, [NR], (F: NR)							
				RT: Adults reported a relative in NH, NR, [NR] (F: NR)							
**Registry**											
1. Frazão SL et al. [MA & TM 14 April 2017]	P (& Signs P), Neglect: Medical and medication	R CS	Registry, Institutional setting or NH, 10 NHs	R: Alleged victim of physical abuse living in a NH 79.7 (NR) [66-107], (F: 79.7%) S: Institutional caregiver NR, [NR], (F: NR)	R: Median 17 months [3 days - 147 months] S: NR	R: ADLs: Severe 33	Registry, NA. Reporting Systems: Items: NR & Forensic medical reports (FMR)	Investigations	120 (10 Yrs.)	R: Suspicion/alleged victim of elder abuse	NR
			R: 1 479 (reports)			-55.90%		(O/D)		Number	
			(3.9%), RR: NA								
2. Friedman L et al. [MA 10 March 2020]	N (clinical signs of neglect)	R CS	Long-term care facilities, 105 LTFs, NFP: 22, FP: 83	R: Inpatients NFP:79.3 (±9.5) (F: 56.9%) FP: 77.7 (±9.3) (F: 63.9%) S: Caregivers	R: NR & S: NR	R: ADL score NFP: 4.9 (±5.5)	Registry, (Inpatient hospital), 11 & Clinical signs of neglect [CSNS] Items: 0 – 60 items	Investigations	60	‘Any’ - One or more clinical signs	Content validity
			R: 430, NFP:61	NR, [NR], (F: NR)		FP: 4.7 (5.4)		(O/D)	(5 Yrs.)		Consensual validity
			FP: 369, RR: NA								
3. Phillips LR & Ziminski C [MA & TM 14 April 2017]	N	R CS	Registry: Complaints	R: Resident NR (±NR), [NR] (F: NR) S: Staff, NR, (±NR), [NR], (F: NR)	R: NR, NR	R: NR	Registry, NA, SC, NR. Registry Arizona Secretary of State Arizona Department of Health Service offices	Allegations of neglect (O)	96	Citation	Interrater reliability
		Exploratory	ALS, 165 (10% Arizona), R: NR, NR		[NR]S: NR				(8 Yrs.)		
			RR: NA%		NR, [NR]						
4. Smith DE et al. 2019, AUS [45] [MA 10 March 2020]	S	R CS	Registry: FMEs from CFM, NR, R: 28, RR: NA	R: Alleged Victim	R: NR	Physical health needs (Y): 11 (39.3%)	Reporting Systems, NA, SC, NR, Clinical case CFM, VIFM, Registry	Alleged Incident	180	Number (alleged incidents)	NR
				NR, [ ≥ 53]	S: NR	Dementia: 17		(O/D)	(15 Yrs.)		
				(F:100%) Median: 83, 80-84: (28.6%) S: Alleged Perpetrator		-73.90%					
				^*^ Direct Care Staff (n = 7) and Medical Practitioners (n = 1), NR							
5. Teaster PB et al. [MA & TM 14 April 2017]	S	CS	Registry NHs NR	R: Older men residing in NHs, 71(NR), [50-93]	NR	NR	Case reports, Registry, NA Reporting Systems: Items: NR & Adult Protective Services (APS) and other regulatory entities from five states & used SASU	Investigations	6	Isolated or ongoing	Reliability testing
			R: 37 Investigation RR: NA, Substantiated: 6/26	(F: 29.7%)				(O/D)		(Y/N) [include substantiate allegations of abuse]	Validity testing
			Perpetrator as staff	S: Facility staff							
			16/24, 75%	NR, [NR], (F:NR)							
6. Teaster PB et al. 2015, USA [40] (Women Only) [MA & TM 14 April 2017]	S	R CS	Registry, NHs, NR R: 64, (40% by staff) RR: NA Substantiated: 20/64	R: Women living in NHs, 81 (NR) [66 -101], (F: 100%) S: Facility staff, NR, [NR], (F:NR)	R: NR & S: NR	R: ADLs Required Assistance	Case reports, Registry, NA, Reporting Systems: Items: NR & Adult Protective Services (APS) and other regulatory entities from five states & used SASU	Investigations	6	Isolated or ongoing	Reliability testing
						45.30%		(O/D)		(Y/N) [include substantiate allegations of abuse]	Validity testing

ADL =  Activities of Daily Living; CRE =  Central Register of Establishments and Enterprises; CFM =  Clinical Forensic Medicine; CNF =  Certified Nursing Facilities; CRE =  Central Register of Establishments and Enterprises; CS =  Cross Sectional; CSNS =  Clinical signs of neglect; DCE=Direct care Employee; DCW=Direct Care Workers; EN =  Enrolled Nurse; F =  Female; FME =  Forensic Medical Examinations; FMR =  Forensic Medical Reports; FP =  For Profit; HRV = Croatia; LTF =  Long-term Facility; LTCF =  Long-term Care Facilities; N =  Nurse; Non-SU =  Non-Specialised Care Units; A =  Not Applicable; NFP =  Non-for-Profit, NR =  Not Reported; RR=Response Rate; Rand =  Random; R =  Resident; R CS =  Retrospective Cross Sectional; RDD =  Random-digit dialling; RN =  Registered Nurse; RR =  Response Rate; RT =  Relatives; S =  Staff; SCU =  Specialised Care Units; Yrs. =  Years; Wks. =  Weeks; UK=United Kingdom.

### Methodological quality assessment

A methodological quality assessment of included articles were independently assessed by three reviewers (TM, MA, and IK) using Boyle et al. (1988) [19] 8-item checklist, designed to evaluate the elements of prevalence studies (S6 File).

## Results

### Study selection

**Fig 1:**
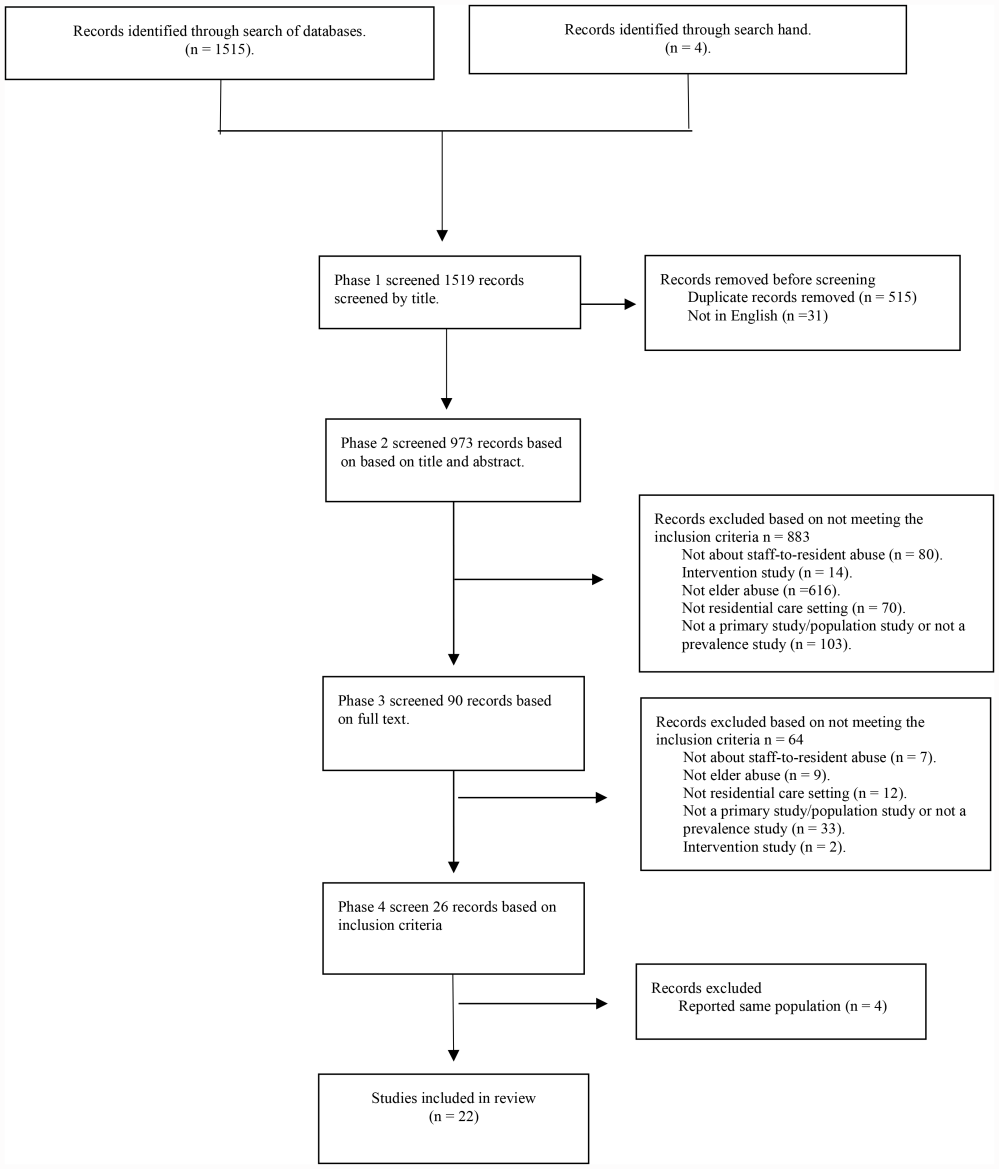
Identification and selection of studies - PRISMA flow diagram.

A total of 1,515 citations were retrieved from the search. Four additional articles were located through hand searching. Duplicates and non-English language papers were then removed resulting in 973 records. Initial screening, against inclusion criteria, of title and abstract, reduced the records to 90. Detailed screening, through full-text review, reduced the records to 44 articles identified as meeting the study criteria. Four papers, by Griffore et al. (2009) [20], Page et al. (2009) [21], Post (2010) [22], Schiamberg et al. (2012) [23] and Zhang et al. (2011) [24], all reported data from the same study population. Griffore et al. (2009) [20] was subsequently retained over the other three, based on a stronger study design including a more defined recall period and a focus on multiple types of abuse. Papers published by Ben Natan et al.(2010) [25,26] and Moore (4) [27–30] used the same population. Ben Natan et al.[26] study examining psycho-social factors affecting elders’ maltreatment in long-term care facilities and Moore’s paper examining observed abuse from two time periods, 2011 to 2013 and from 2015 to 2019 with prevalence data were chosen [31,32]. While other studies did not provide prevalence data of abuse [33,34] or examined perception of elder abuse and neglect among nursing staff working in a hospital [35]. The final study cohort comprised 22 studies (Fig 1) [15].

### Study characteristics

Country settings varied, with eight from the United States of America [USA] [20,36–42], two from Israel [25,43], two from Australia [44,45], nine from individual European countries [46–53] and two from the United Kingdom [31,32].

Similarly, the studies were methodologically diverse, with 16 cross-sectional, 13 studies collected abuse data from staff, with the majority using surveys [36,37,45,47,48,53] or questionnaires [25,46,50,52]. Three studies utilised a mixed methods approach to distribute a staff questionnaire and interview [31,32,42], however for the purpose of this review we only included questionnaire data. A total of three studies were reviewed which collected data from residents. Residents were interviewed in two studies [48,51], while one study by Cohen et al.(2010) [43], interviewed and collected data using participants’ health profile to determine signs of abuse from risk indicators of maltreatment or abusive acts.

The remaining cross-sectional study randomly selected ‘family members’ from the general community to participate in a telephone interview [21] and one from a community registry [42]. The five retrospective cross-sectional studies used one or more existing abuse reporting systems or registries [38–40,49]. The following study characteristics are arranged based on persons reported abuse, from highest contact with resident (staff) to least contact with resident (registries) to examine recruitment methodology and study participant details (Table 1).

#### Recruitment and participant characteristics – staff.

The 13 studies that recruited staff [26,32,33,37,38,43,46–49,51,53,54] did so by selecting institutes or homes ranging from 2 [43] to 1,600 [47]. Of those who recruited staff cohorts ranged from 53 [46] to 7,000 [37,38]. Twelve studies reported a response rate, ranging from 15% [43] to 92% [26] (Table 1). Staff participant characteristics were reported in eleven studies; ten reported the majority being female (>82%) [26,37,38,43,46–49,51,53,54]; with an age range between 16 to 74 in seven studies, [53] and 13.7 mean years experienced as reported in five studies [26,46,51,53,54]. Current nursing home experience ranging from 1.1 [36] to 8 years [53]. Of the thirteen studies that utilized reported abuse by staff, four also collected data on one or more resident characteristics [43,47,49,53] (Table 1).

#### Recruitment and participant characteristics – residents.

Three studies [44,49,52] examined older adult abuse as reported by residents. One study selected 10 ‘nursing homes with 640 eligible ‘nursing home residents invited 200 to participate, with 82% doing so [52], while another study selected 24 ‘senior homes’, screened 1,807 ‘clients’, with 27% meeting the study criteria [49]. The third study collected data from 71 admitted ‘inpatients’ from ‘elderly homes’ or ‘nursing homes’ [44]. No response rates were recorded. The majority of the participants were female (>65%) [44,49,52] with a mean age of 82 (±7.5) (range: 60 – 99) [44,49,52]. Activities of Daily Living (ADL) was used to report resident dependency levels and years of residency was reported in one study as ‘length of stay’, with two-thirds reporting less than four years [49]. No staff characteristics were reported in the studies recruiting residents (Table 1).

#### Recruitment and participant characteristics – relatives.

Relatives were recruited in a telephone survey [21]. There were 450 participants from the general community who had ‘a family member’ ‘receiving long-term care services’. No response rate was recorded. The only study characteristics collected were description of residents, as the ‘family member’ being female (73%). No staff characteristics were reported (Table 1).

#### Recruitment and participant characteristics – registry.

Six studies utilized existing registries to report institutional abuse examining sexual abuse over a six-month period [41,42], complaints of neglect over an eight-year period [40] or as an admitted patient due to neglect over a five-year period [39]. The two studies examined forensic medical reports for incidences of ‘female sexual abuse’ [45] or ‘abuse’ [50] from ‘nursing homes’ or ‘institutional settings’ over a 10 [50] to 15 [45] year period resulting in small prevalent cohorts, ranging from 28 [45] to 59 [50], respectively (Table 1).

Of the six studies, three reported a mean age of 79.7 years [39,41,50]. Resident’s dependency was reported as an ADL status, ‘being mildly to totally dependent’(62%) [41], a category [‘mild/moderated’ or ‘severe’ (‘loss of autonomy’ [highest, severe 56%])] [50], a gradient [‘ability to function independently’ 0 – 10 scale (aggregate mean 4.8 [Barthel Index])] [39] or as ‘dependency or require assistance with ADLs’ (18%)] [45]. Years of residency was only reported in one study, at the time of registered abuse, with a median of 17 months [49] (Table 1).

#### Methodology instruments used to measure older aged abuse.

Overall, we identified 16 instruments used to measure older aged abuse in long term institutes over the last 18 years. In Table 1, we constructed columns examining the methodological approaches, based on who reported the abuse (highest contact with resident, staff to lowest registries) with the following methodological features such as 1) method of administering data (such as mode, distribution and collection) 2) number of items and name of the identified instruments [including source], 3) observed and/or experienced abuse with type of abuse reported 4) recall period and 5) validity of tool. In the S3 Table, we have provided a more in-depth analysis to examine the homogeneity of the commonly defined types of abuse (as defined by WHO).

#### Instruments used to measure older age abuse - as reported by staff, and/or residents.

Of the 16 instruments, 11 were used to measure abuse as observed and/or experienced by the staff member, resident or relatives. The review found the three most commonly used tools measured staff abuse; Malmedal et al’s 42 acts of inadequate care instrument (2009) [47,52,53]; Castle’s (2012) 28-item questionnaire measuring how often staff observed and/or perpetrated abuse [37,38,48] and Drennan et al.(2012) [50,53,54] national survey on interactions and conflicts within nursing home settings. There were variations among these instruments ranging from modes of delivery, either presented as a questionnaire or survey, with differences in definition and types of abuse and discrepancies in recall periods.

Malmedal et al.(2009) [53] original 42-item questionnaire was used to measure staff ‘observed’ or ‘committed’ ‘physical, psychological (emotional), financial and neglect’ acts (unintentional and intentional acts) of inadequate care’ within a four-week recall period, using a four-point Likert-type frequency scale ranging from ‘never’ to ‘more than once a week’ was used in two other studies. This questionnaire has been tested for face validity only, indicating the tool was easy to follow and comprehensive, evidence pertaining to the other items of validity were not reported.

Habjanič and Lahe (2012) [52] further modified Malmedal’s (2009) [53] question which asked ‘residents’ face-to-face if they had ‘ever experienced’ ‘mental’, ‘physical’ and ‘financial abuse’ using a ‘six months’ recall period to record the ‘number of incidences’, rather than using the Malmedal et al.(2009) [53] Likert scale (Table 1). In 2017, Blumenfeld Arens et al.[47] used Malmedal et al.(2009) [53] to ask staff if they only ‘observed’ ‘elder abuse’ (not as the original definition, ‘inadequate acts’ of ‘physical, psychological (emotional) and neglect, but not financial abuse, using with the same recall period and Likert-type frequency scale. None of the studies measured correlation ecoefficiency.

Castle and Beach’s (2013) [38] 46-item questionnaire measured the ‘number’ of times staff ‘observed’ ‘physical’, ‘psychological (verbal)’, ‘financial (material exploitation)’ or ‘sexual abuse’ in ‘last three months of their prior place of employment’ and was used again in a study by Castle the following year [37]. The questionnaire has been tested for face and content validity (using the Fleisch–Kinkaid Scale), indicating this tool is measuring the degree which abuse is measuring abuse accurately. Recently, Botngård et al.(2020) [48] changed Castle’s questionnaire to examine staff’s incidences of ‘observed’ and ‘perpetrated’ abuse, measuring additional types of abuse, ‘overall’ and ‘neglect’, within their ‘current place of employment’ in the ‘last 12 months’ All studies utilized the original frequency using a three-point Likert-type scale as ‘never’,’once’ or ‘repeatedly’ (Table 1). None of the studies reported on correlation ecoefficiency.

Gil & Capelas (2022) [51], and Neuberg (2017) [54] utilised the long-established questionnaire by Drennan et al.(2012) [55], a 25-item national survey of staff-resident interactions and conflicts within residential care settings. Between the two papers, there were variations with types of abuse and whether it measured witnessed [54] and/or committed [51] abuse. In Neuberg et al. [54] study, the survey was pretested in a validation pilot study and achieved a reliability coefficient was >  0.7, deeming the instrument to be reliable. Overall, there is still some heterogeneity among these instruments, they are still in their early constructs, more studies and methodology testing are required conducted to validate these instruments. See Table 1, for further details.

#### Instruments using data registries to measure older age abuse.

The remaining six studies utilized government registries or databases. Four studies utilized existing government registries such as the Registry Arizona Secretary of State & Arizona Department of Health Service offices [40] or the Adult Protective Services (APS) (National Adult Protective Services Association [NAPSA], 2021) (3) in conjunction with a survey (Sex Abuse Survey [SASU]) [41] and/or with hospital records with the use of Clinical Signs of Neglect Scale (CSNS) [39,41,42] to report ‘isolated or ongoing’ investigation of ‘citations and allegations’ [40] or a ‘suspected, reported, unsatisfactory, partial or substantiated resolution case of abuse’ [41,45,50] or used the to identify ‘clinical signs of elder mistreatment or elder neglect’ [39]. While two studies utilized clinical forensic medicine reports [45,50] of ‘current or past medical observations and/or victim complaints of suspicion of physical or psychological abuse’ [50] or ‘alleged incidence of sexual assault among women only’ to report incidences of abuse [45]. These studies varied with recall periods ranging from six months [41,42] to 15 years [45]. Some of these studies required validated professional staff to perform examinations [50], a consortium of experts to develop clinical validated scales [39,41,42] or independent research reviewers [44] ensuring reliability, validity and reliability of findings, however, registers are commonly known for their practical limitations such as incompleteness or inaccuracy with data collected and difficulties with data dredging (Table 1).

### Impact of methodology on the results

#### All abuse.

Out of the 22 studies, ten studies measured the overall incidence of abuse (measuring one or more types of abuse as defined by WHO) [26,32,33,43,44,48–51,53] (Table 1), with the highest overall prevalence reported over a four week period reported in one study, 91% ‘observed’ abuse by staff, while ‘committed’ abuse by staff was at 87% [53]. Two studies reported abuse by staff over a 12-month period resulted in lower rates of ‘observed’ abuse ranging from 55% [51] to 76% [48] and for ‘perpetrating’, from 54% [26]to 60% [48].

Two studies reported by ‘residents’ overall abuse over a 12-month period, retained lower rates, than above. ‘Experienced’ abuse ranged from 11% [49]to 31% [44], while ‘observed’ was at a lower rate of 5% in one study [49]. No studies examined overall abuse reported by relatives or the community via a registry. Five studies (23%) reported all five types of abuse as defined by WHO [21,26,44,48,51] (See Table 1). The following sections will examine prevalence based on types of abuse as defined by WHO, physical, psychological, financial, sexual and neglect.

#### Physical abuse.

The most commonly measured form of abuse was physical abuse, also defined as ‘physical violence’ [26], ‘mistreatment [21], ‘maltreatment’ [44] or ‘acts of physical character’ [53], measured in 15 studies [21,26,32,37,38,43,44,47–54]. An accumulation of 81 items were identified to describe acts of physical abuse, with each study using three [47] to 11 [44] items to describe physical abuse. The most commonly used verbs to describe physical abuse was ‘hitting’ (8) [21,37,44,48,49,52] or ‘kicked’ (7) [21,37,48,49,52], with variations in definition, recall periods and persons reported. One study relied on staff to define physical abuse [32] or did not disclose items measured [33] (S3 Table: Definitions of types of abuse).

The highest rate of physical abuse reported was ‘witnessed’ by staff (44%), in the act of ‘restraining/hold back a resident’ ‘over a recall period of four weeks’ [53], and the highest ‘committed’ abuse was 33% from the same act of ‘restraining/hold back a resident’ as reported in the same study. When the same questionnaire was used in Blumenfeld Arens et al. [47] in 2017, the study questionnaire, measured witness physical abuse over a 4-week period, resulting in a lower rate of 1.4%.

Studies examining physical abuse over a 12-month period, Gil and Capelas (2022) [51] and Neuberg et al. (2017) [54] using the same questionnaire [54], resulted in different levels of physical abuse by staff. Neuberg et al. (2017) [53] reported over 12 months, 42% of staff observed ‘force feeding the resident’ in the last 12 months, whereas Gils and Capelas (2022) [51] recorded 14% observed staff committing ‘at least 1 of the 6 behaviours of physical abuse’. The remaining studies utilised various measurement tools, with the same recall period of 12-months resulting in observed rates ranging from 6% [32] to 30% [49], while committed abuse were even lower ranging from 1.7% [32] to 12.3% [26].

Three studies reported physical abuse reported by residents either in the last six [52] to 12 months [44] resulted in lower rates of ‘observed’ abuse, from 1% [49] to 2% [49] and 8% for ‘experienced’ abuse [44]. Cohen’s study [44] found only three residents attained a score of three or more on the signs of physical scale. Compared to this, relatives reporting abuse in telephone interviews in the last 12 months had higher rates of abuse at 74% [21], while physical signs or evidence of physical abuse from forensic medical reports (FMR) from registries, were lower at 55 cases over a 10-year period [49], however these tend to be extreme cases of abuse (S3 Table).

#### Psychological abuse.

The second most common measured form of older age abuse in long term institutes was psychological abuse. Fourteen studies [26,32,37,38,43,44,47–49,51–54] addressed psychological abuse. Three studies defined this type of abuse as ‘psychological abuse’ [32,44,48,49,51] while the remaining six defined as ‘emotional’ [21,43,47,52], ‘mental abuse’ [26,53] or as a combination of ‘psychological and verbal abuse’ [37] or ‘emotional or psychological and verbal mistreatment’ [21].

There were in total 47 items, with each study using three [47]to 14 [49] items to classify psychological abuse. The most common terms used to describe psychological abuse were of ‘intent’(5) [49] or ‘threat’(4) [37,49,52,53]. A total of four studies did not disclose items or descriptions of types or examples of abuse asked [26,43,54] (S3 Table).

Psychological abuse was reported by staff (11) [26,32,37,38,43,47–49,51,53,54], residents (2) [44,52] and relatives (1) [21]. The highest rates of ‘observed’ and ‘committed’ acts of psychological abuse was ‘entering a room without knocking’ of abuse by staff, 64% of staff committed the act, while 84% observed other staff in the last four weeks [53]. Three studies examining psychological abuse ‘committed’ by staff over the last 12 months found higher incidents ranging from 23% [26] to 46% [49], with variations in instruments utilized to measure this form of abuse. ‘Observed’ abuse by staff was reported in five studies, with incidents ranging from 30% [49] to 62% [21], with variation in instruments used making it difficult to provide an average rate. Two studies utilised Dennan et al. [54] instrument however there was a 20% difference between the act of shouting at resident in anger [50] (33% [50] from 16 care home settings versus 55% from the nursing home and extended care units [54]).

Residents reported ‘experienced’ psychological abuse ranged from 10% over a 12-month period [49] to 56% [52] over a six-month period, however, reported ‘observed’ abuse was lower at 4% [49]. Uniquely, Cohen et al. (2010) [44] reported distribution of disclosed abuse and found “very low complaints for psychological abuse” (13%). Telephone interviews among family members (relatives) reported 84% ‘observed’ ‘verbal mistreatment’ by nursing staff ‘in the previous year’ [21]. No studies measuring psychological abuse used registries. All studies examined specific psychological acts, making it difficult to aggregate the incident rate due to variations as shown above (S3 Table).

#### Financial abuse.

Eleven studies defined financial abuse either as ‘material exploitation’ [21,37,38,48] and/or ‘financial exploitation’ [26,44], ‘financial abuse’ [32,51,52], ‘acts of financial character’ [21,48,53]. An accumulation of 24 items was identified to describe acts of financial abuse, with each study using one [53] to seven [44] items to describe financial abuse. Most common term used to describe financial abuse was ‘signing documents’ (6) [37,44,48,52] (S3 Table).

Most of the studies examining the rates of financial abuse were reported by staff (8) [26,32,37,38,43,48,51,53], followed by residents (2) [44,52]or relatives (1) [21]. The highest level of financial abuse reported in this review were observations of staff from relatives of older adults residing in nursing homes, 71.9% [21]. This was followed by reported ‘experienced’ financial abuse by residents in one study, at 32.8% [52] over the last 6 months. Lower rates of financial abuse were reported by staff, for ‘observed’ incidents ranged from 2.1% [48] to 3.3% [51] in care and nursing homes.

Staff reporting ‘committing’ financial abuse were at a lower rate 0% [53] to < 1% [26,48] over the last four weeks to 12 months, while two studies examined staff ‘observed’ financial abuse found 10%, of staff took ‘assets’ from nursing home residents or ‘destroying belongings’ of resident residing in assisted living institutes, 26% [38]. Interestingly Castle’s questionnaire used in two studies, in two similar settings and recall periods, found the incidents from ‘taking residents assets’ were similar, 10% [37] versus 11% [37]. No studies measuring financial abuse used registries (S3 Table).

#### Sexual abuse.

Eleven studies reported the prevalence of sexual abuse, described either as abuse [21,26,32,38,41–46,48,49,51] with variation in definition of this form of abuse ranging from as an act of ‘assault’ [45], ‘misconduct’ [21], ‘violence’ [26], ‘unlawful or unwelcome sexual behaviour’[46] or ‘sexual nature without consent’ [51], to an outcome of signs of ‘forensic evidence’ [42] or ‘victimization (women)’ [41].

Number of items describing sexual abuse ranged from one [51] to 11 items [41]. Among the eleven studies, in total were 34 items that identified abuse including, as an act of exposure to (4) [37,41,42] (hands off) to oral-genital contact (3) [37,41,42] (hands on). Evidence of signs of sexual abuse included a torn underwear to infected [44].

Most of the studies relied on reports by staff (4) [26,37,48,49], or registries (3) [41,42,46], followed by direct reporting from residents (2) [44,49]and one by relatives [21]. Reports from relatives had the highest reported level of sexual abuse at 40% [21]. Registries reporting an incidence of sexual abuse performed by staff ranged from 15.6% to 25% [41,42,45] however these cases were over a ten-to-15-year period. The lowest reported incidences of this type of abuse were reported by staff as ‘observed’ resulted in ≤  7% or ‘committed’ < 1% [26,32,37,38,43,48,49,51], while two studies reporting no sexual abuse reported by residents [44,49]. There were two studies showing some consistency with findings, utilising the same questionnaire in different institutionalised settings, found staff observed 69 nursing home staff and 61 assisted living staff ‘exposed private body parts to embarrass resident’ in the last three months [37,38].

#### Neglect.

Similar to psychological abuse studies, neglect is equally the second highest form of abuse investigated in this review among older adults residing in long term institutions [21,26,32,39,40,43,44,47–51,53,54].

The definition of neglect varied with 3 [40] to 11 items [39] describing these acts from ‘physical and mental neglect’ [26], to ‘clinical sign of neglect’ [39,44,50], or collectively categorised as ‘personal, environmental, medical’ [40] to specific items described care neglect such as ‘not changing the position of bedridden person’ or ‘ignoring resident when they called’ [49,51,54]. Only two studies utilised the same instrument to measure neglect [37,38]. Four studies did not provide or specify items that were measured for this type of abuse [26,43,54].

Neglect ‘observed’ by relatives retains the highest rate of failure to provide basic needs to residents (86.9%) [21]. Four studies reported neglect ‘committed’ by staff over the last 12 months, with results varied from 1% [49] to 46.9% [48], compared to nine studies reporting ‘observed’ acts of neglect ranging from 9% [49] to 57.8% [48]. These variations are due to different instruments and definitions used to measure neglect. Surprisingly, four studies used the same instruments, however disseminated findings differently, with one study reporting if ‘observed’ or ‘committed’ one of the ten items listed for neglect, while the other reported 10 items distinctly with respected incident rates [47,51,53,54].

The highest prevalence of neglect was 24%, attained from the face-to-face interviews conducted by hospital staff [44] among inpatient residents, while another study when interviewing residents on ‘observed’ or ‘experienced’ neglect conducted in facilities were ‘unmentioned’ [49]. Registries reported 20% of severe cases of neglect, however again, this was over a 10 or more-year period [39,50], while another study reported a total of 1,196 total neglect allegations, with 535 substantiated, over an eight year period, making it difficult synthesis findings [39]. Other abuse items not classified by WHO are also included in the S3 Table.

### Methodological quality assessments

Studies were assessed and ranked by methodological score and categorized according to their study design and sampling (Table 2); using an eight-item methodological scoring standardized checklist [20]. Two independent reviewers scored a total of 88 items and agreed on 82 (93%) (κ 0.90 (95% CI 0.88 to 0.99), p < 0.001) meaning there was a high agreement. The only minor discrepancy was from the interpretation of validated measurement tools. The representation of samples was at times not reported (85%), with no studies examining non-respondents and five studies (36%) reporting response rates [26,37,48,53]. Only one study accounted for sampling design in their analysis [47], while all studies did not report confident intervals for prevalence rates (item 8). A total of four studies (29%) achieved a total score above 5 [47,48,52,53], with scores ranging from 1/8 to 6/8.

**Table 2:** Methodological quality of studies.

	Author, Year, Country	Mode of Recruitment	Q1	Q2	Q3	Q4	Q5	Q6	Q7	Q8	Total Quality Score/8
**Number**	**NURSING HOMES**										
**1.**	Botngård A et al,2020, NO [48]	NHs	Y	Y	0	Y	Y	Y	Y	N	**6**
**2.**	Habjanič A & Lahe D, 2012, SI [52]	NHs	Y	Y	Y	Y	Y	Y	N	N	**6**
**3.**	Blumenfeld Arens O, 2017, SW [47]	NHs	Y	Y	N	Y	N	Y	Y	N	**5**
**4.**	Malmedal W et al, 2009, NO [53]	NHs	Y	Y	N	Y	Y	Y	N	N	**5**
**5.**	Buzgová, R & Ivanová, K, 2011, CR [49]	NHs	Y	Y	N	Y	Y	N	N	N	**4**
**6.**	Castle N, 2012, USA [37]	NHs	Y	Y	N	Y	Y	N	N	N	**4**
**7.**	Ben Natan M et al, 2010, IL [26]	NHs	N	Y	Y	Y	N	N	N	N	**3**
**8.**	Griffore RJ et al, 2009, USA [21]	CATI	Y	Y	N	Y	N	N	N	N	**3**
**9.**	Neuberg M et al, 2017, HRV [54]	NHs & ECUs	N	N	N	Y	Y	Y	N	N	**3**
**10.**	Smith DE et al, 2022, AUS [46]	Registered subscribers to resource on resident safety	Y	N	N	Y	N	N	N	N	**2**
**11.**	Gil AP & Capelas ML, 2022, PT [51]	NHs	0	0	0	1	0	0	0	0	**1**
	**ASSISTANT LIVING**										
**1.**	Castle N & Beach S, 2013, USA [38]	Professional Registration Nurse Aides	1	1	0	1	1	0	0	0	**4**
**2.**	McCool JJ et al, 2009, USA [43]	ALF & ECUs	0	0	0	1	0	0	0	0	**1**
	**CARE HOMES**										
**1.**	Moore S, 2016, UK [32]	CHs	0	0	0	1	0	0	0	0	**1**
**2.**	Moore S, 2020, UK [33]	CHs	0	0	0	1	0	0	0	0	**1**
	**REGISTRIES**										
**1.**	Teaster PB et al, 2007, USA [42]	RG	1	0	0	1	1	1	0	0	**4**
**2.**	Teaster PB et al, 2015, USA [41]	RG	1	0	0	1	1	1	0	0	**4**
**3.**	Phillips LR & Ziminski C, 2012, USA [40]	RG from ALFs	1	0	0	1	0	1	0	0	**3**
**4.**	Smith DE et al, 2019, AUS [45]	RG(s)	1	0	0	1	0	0	0	0	**2**
**5.**	Frazão SL et al, 2015, PT [48]	RG	1	0	0	0	0	0	0	0	**1**
	**HOSPITALS**										
**1.**	Cohen M et al, 2010, IL [44]	Hosp	1	0	0	1	1	1	0	0	**4**
**2**	Friedman L et al, 2017, USA [39]	Hosp	1	1	0	1	1	0	0	0	**4**

ALFs = Assistant Living Facilities; CATI =  computer-assisted telephone interviewing; ECUs = Extended Care Units; Hosp =  Hospitals; NHs =  Nursing Homes; RG =  Registries, Y =  Yes; N =  No; U =  Unclear; NA =  Not Applicable.

## Discussion

The main aim of this review was to comprehensively illustrate and critique methodologies used within the field. We identified heterogeneity in how researchers employed, variations with sampling techniques, data collection procedures who reported abuse, measurement tools and recall periods from all potential sources of reported abuse in long-term care institutions. There was little comparability between studies and variable study quality made it difficult to synthesise findings, and not possible to establish the prevalence of abuse rates. Furthermore, we also found the quality of studies varied significantly, with no consistency.

Similar to previous reviews, we found most articles focus on all types of abuse rather than just physical and psychological abuse [5,6,11,12]. Research in this field has undergone a notable shift, focusing predominantly on the clearly defined parameters of abuse outlined by the WHO in 2002. We found the majority of studies examine abuse from the staff perspective, with few reporting from residents, relatives and community members[6,11]. Researchers have utilised study designs to include not only staff reporting abuse but other sources such as residents incorporating clinician signs of abuse, relatives and the general public. Consistent with findings from other reviews, we report that relatives, followed by staff typically report the highest incidence of observed abuse, whereas resident reports the lowest abuse [6,11,12]. Furthermore, measurement tools used via registries often yield lower prevalence rates due to their tendency to report extreme cases of physical signs and reported abuse, however, this tends to be limited to physical or sexual abuse and/or neglect. Despite this, these tools may offer valuable insight into measuring abuse, as they provide concrete means to validate abuse occurrences.

Among the 22 studies in the review, there was no consistency in presenting the study’s participants or cohort characteristics, making it difficult to conduct comparability or understand individual study’s generalisability. The majority of cohort studies described their participant’s characteristics, either staff, residents or community members, using one characteristic. This point reveals there is no agreement, within or across countries, about what and how characteristics should be reported.

There was also no consistency across the 22 studies in methods and measurement tools used for investigating staff abuse among residents. Only six [38,47,48,51,52,54] of the 22 studies had used three previously developed methodologies [37,53] to measure older adult abuse, however, modifications were made to these original questionnaires, impacting the ability to compare findings. There were variations with recruitment methods resulting in different sample sizes and a lack of consistency in who was reporting the abuse, concluding in differences in findings. Only two studies utilised an independent researcher to personally distribute the questionnaire to staff [26] or interviewed residents face-to-face as an inpatient admitted to hospital for reasons unrelated to an incident of abuse [44] avoiding explicit bias in data analysis. Furthermore, only one study reported a prevalence of ‘self-reported’, ‘observed’, ‘committed’ or ‘experienced’ forms of older abuse by both staff and residents [49]. Study designs that focus on staff or residents reporting abuse to other staff members or facility managers, deter disclosure in their responses or create stigma and blame among staff who have witnessed or committed abuse, resulting in underestimated rates of abuse [48,53]. Anonymity of those who distribute the survey, conduct interviews or examinations will reduce bias and improve the reliability of the study’s findings [26,37,44]. Inconsistencies observed in elder abuse research arise from multifaceted reasons. Heterogeneous definitions of abuse employed by researchers contribute to disparities, variations in reporting sources, from staff to relatives and researchers utilizing different definitions of abuse, with different recall periods adds further complexity to the synthesis of these findings.

It is evident that despite an increased interest in older adult abuse, as previous authors have cited, there has been minimal progress in standardising abuse measurements nationally nor globally [14]. This point highlights the unmet need to generate a robust standardized prevalence measurement tool of all types of older abuse, for use at national and global levels [7]. Instead of developing a modified questionnaire or survey, future research should focus on external validating current questionnaires.

Finally, the overall methodological assessment of the cohort of studies was poor, with only four of the 22 studies, meeting the standard expected by Boyce’s [20] prevalence study criteria. The individual studies themselves are accreditable. Heterogeneity in methodology is not valid or creditable to draw conclusions in the understanding prevalence of older adult abuse on a national nor global level. Boyce’s tool, the most generic one available, was not designed for this field and may therefore have limited the findings. A recently published protocol paper has outlined plans for a forthcoming systematic review that will investigate the psychometric properties of instrument designs aimed at assessing elder abuse prevalence in both community and institutional settings [55].

From this review, the most appropriate methodological choice for measuring older adult abuse in institutional settings would be Malmedal’s et al. (2009) [53] original 42-item questionnaire. This is based on limited evidence, a high-quality assessment score and repeatability of the measurement tool in three studies [45,50,51], exhibiting a close to consistency in results. Thus, the analysis has revealed that to improve the knowledge base, there is a need for testing consistency in methods and measurement tools used for investigating staff abuse among residents. This includes greater participation from all stakeholders in research [48], and a standardised, comprehensive set of tools and data elements to be utilised. The WHO definitions provide a basis upon which these resources can be established [51]. This approach will enable accurate measurement of abuse and promote construct validity and reliability measurement tools on abuse of older adults [56,57]. The proposed resources will assist in implementing effective workplace management programs to tailor associated risk factors of abuse within institutionalised care. These resources could be developed by a global consortium of experts and patient representatives, similar to internationally established methodologies in other health fields, including clinical and psychological topics [58].

Additionally, there is a need to establish a methodological quality assessment tool specific for institutionalised care to determine the level of quality of evidence. This work could take direction from that by Giannakopoulos et al. (2012) [59] and Shamliyan et al. (2010) [60] who developed instruments measuring the quality of studies examining the prevalence of disorders and diagnostic protocols or rates and risk factors for diseases. Gerontology researchers can further develop the evidence base by undertaking translational research projects.

As a key step towards improving the evidence base and establishing standardised research tools identified above, we have developed the Aged Care Abuse Research Checklist (ACARC) (S2 Table). This tool has been derived from the 22 empirical studies’ key strengths [20,25,31,32,36–53] and is designed to improve the methodological quality and research rigor for future studies. The ACARC comprises 11 points covering study design (2) [37,44,47], methodology (6) [25,38–44,47,49,61,62], results (2) [8,20] and publication (1). The widespread use of ACARC can promote researchers’ engagement in collecting prevalence data on aged care abuse on national and international scales. With consistent and reliable data obtained through standardized research measurement tools on older adult abuse, policymakers can gain deeper insights into the prevalence and nature of elder abuse, as well as the quality of care provided in various institutions, organizations or facilities. This knowledge could also develop evidence-based policies tailored to address specific areas of improvement in the aged care sector, such as educate and improve staff’s understanding and identification of abuse behaviours [63,64] and provide the broader industry policy direction [9]. All outcomes which will contribute to improvements in residents’ quality of life, safety and quality of care, and staff wellbeing – together which contribute to the quadruple aim in healthcare [65].

## Limitations

A limitation of this review was that it did not include studies examining residential special units. These environments were excluded because of their different clinical focus and unique challenge in involving residents in research. Nevertheless, the decision may have potentially excluded methodological tools measuring higher abuse rates other than indicated in this review. There is a need to conduct a specialized review and analysis for these institutionalized settings, as these groups have different needs and demands or present findings of these subgroups within articles [43,46,47].

## Conclusion

The review examined research methodologies used when investigating abuse within the aged care field. Relatives and staff typically report highest abuse rates, while residents report fewer incidents, even with fewer incidents of observed abuse. Registries tend to capture extreme cases, resulting in lower reported prevalence rates, particularly of physical or sexual abuse and neglect. Physical abuse was the most reported, with 81 different descriptors identified and varying recall periods. Staff witnessing abuse ranged from 44% over four weeks to as low as 1.4% over 12 months, posing challenges for data interpretation. The review identified a heterogeneity of definitions of abuse, variation of who reported abuse, lack of agreement on measurement tools and recall periods, and variable study quality. To develop evidence-based methodology there is a need for standardised, comprehensive resources for the field. Ideally, a global consortium could be established to determine how to consistently define, accurately measure, report, analyse, and respond to abuse. The Aged Care Abuse Research Checklist (ACARC) (S2 Table) was developed from the review as a first step towards achieving this outcome. Doing so will normalise processes within organisations and the community, allowing early interventions to change practices and reduce the risk of recurrence. These arrangements will improve resident quality of care and workplace cultures.

## Supporting information

S1 Checklist PRISMA Checklist 2020 (Word). (DOCX)

S2 Checklist PRISMA Abstract Checklist 2020 (Word). (DOCX)

S1 File Prospero Registration Systematic Review Protocol (PDF). (PDF)

S2 File Selected electronic databases (Word). (DOCX)

S3 File Search terms and strategy (Word). (DOCX)

S1 Table Study eligibility using PICOTS framework (Word). (DOCX)

S4 File Reasons for inclusion and exclusion of studies from the systematic review (Excel). (XLSX)

S5 File Data extraction template variables information eligible studies (Word). (DOCX)

S6 File Methodological Quality Criteria List (Word). (DOCX)

S2 Table Aged Care Abuse Research Checklist (ACARC) (Word). (DOCX)

S3 Table Definitions of Types of Abuse (Word). (DOCX)
