# Supplementary material for: Global approaches to older abuse research in institutional care settings: A systematic review
Source: PLoS One. 2025 Mar 10;20(3):e0290482. doi: 10.1371/journal.pone.0290482 (PMC11892848; doi:10.1371/journal.pone.0290482)
Supplement: S1 File — (PDF) [file pone.0290482.s003.pdf]

Systematic review

*Fields that have an **asterisk (*)** next to them means that they **must be answered.** **Word limits** are provided for each section. You will be unable to submit the form if the word limits are exceeded for any section. Registrant means the person filling out the form.*

3. * Anticipated or actual start date. [1 change]

Give the date the systematic review started or is expected to start.

08/11/2017

4. * Anticipated completion date. [4 changes]

Give the date by which the review is expected to be completed.

31/05/2020

5. * Stage of review at time of this submission. [6 changes]

This field uses answers to initial screening questions. It cannot be edited until after registration.

Tick the boxes to show which review tasks have been started and which have been completed.

Update this field each time any amendments are made to a published record.

The review has not yet started: No

Review stage	Started	Completed
Preliminary searches	Yes	Yes
Piloting of the study selection process	Yes	Yes
Formal screening of search results against eligibility criteria	Yes	Yes
Data extraction	Yes	Yes
Risk of bias (quality) assessment	Yes	Yes
Data analysis	Yes	Yes

Provide any other relevant information about the stage of the review here.

11. * Review team members and their organisational affiliations. [2 changes]

Give the personal details and the organisational affiliations of each member of the review team. Affiliation refers to groups or organisations to which review team members belong.
NOTE: email and country now MUST be entered for each person, unless you are amending a published record.

Dr Maria Agaliotis. Australian Institute of Health Service Management, University of Tasmania
Professor Ilana Katz. Social Policy Research Centre, UNSW
Mr Tracey Morris. Student, UNSW

14. Collaborators.

Give the name and affiliation of any individuals or organisations who are working on the review but who are not listed as review team members. **NOTE: email and country must be completed for each person, unless you are amending a published record.**

34. Reference and/or URL for published protocol. [2 changes]

If the protocol for this review is published provide details (authors, title and journal details, preferably in Vancouver format)

Yes I give permission for this file to be made publicly available

37. Details of any existing review of the same topic by the same authors.

If you are registering an update of an existing review give details of the earlier versions and include a full bibliographic reference, if available.

38. * Current review status. [4 changes]

Update review status when the review is completed and when it is published.
New registrations must be ongoing so this field is not editable for initial submission.

Review_Completed_not_published

40. Details of final report/publication(s) or preprints if available. [1 change]

Leave empty until publication details are available OR you have a link to a preprint (NOTE: this field is not editable for initial submission).
List authors, title and journal details preferably in Vancouver format.
