# Supplementary material for: Global approaches to older abuse research in institutional care settings: A systematic review
Source: PLoS One. 2025 Mar 10;20(3):e0290482. doi: 10.1371/journal.pone.0290482 (PMC11892848; doi:10.1371/journal.pone.0290482)
Supplement: S2 File — (DOCX) [file pone.0290482.s004.docx]

# S2 File. Selected electronic databases

- Cumulative Index to Nursing and Allied Health Literature (CINAHL)(EBSCO interface, 1981 onwards);
- Cochrane Database of Systematic Reviews (OVID interface, 1999 onwards);
- EMBASE (OVID interface, 1946 onwards);
- GlobalHealth (OVID interface, 2000 onwards);
- MEDLINE (OVID interface, 1946 onwards);
- PsycINFO (OVID interface, 1806 onwards);
- PubMed (NCBI interface, 1996 onwards);
- Scopus;
- Social Work Abstracts (OVID interface, 1968 onwards);
- Web of Science up to April 2020.
