# Supplementary material for: Global approaches to older abuse research in institutional care settings: A systematic review
Source: PLoS One. 2025 Mar 10;20(3):e0290482. doi: 10.1371/journal.pone.0290482 (PMC11892848; doi:10.1371/journal.pone.0290482)
Supplement: S3 File — (DOCX) [file pone.0290482.s005.docx]

**S3 File. Search terms and strategy**

Search strategy used on Medline Ovid 4 June 2024

1 (Geriatric* or Elder* or old-age* or pensioner*).mp. [mp=title, abstract, original title, name of substance word, subject heading word, floating sub-heading word, keyword heading word, organism supplementary concept word, protocol supplementary concept word, rare disease supplementary concept word, unique identifier, synonyms] (447101)

2 (Aging or aged or senior or old*).mp. [mp=title, abstract, original title, name of substance word, subject heading word, floating sub-heading word, keyword heading word, organism supplementary concept word, protocol supplementary concept word, rare disease supplementary concept word, unique identifier, synonyms] (7364936)

3 exp Aged/ or exp geriatrics/ (3533893)

4 1 or 2 or 3 (7435163)

5 ((mental* or physical or verbal or emotional or financial or sexual or psychological or material or elder) adj2 (harm or abus*)).ti,ab. (28769)

6 (neglect* or ill-treat* or maltreat* or mistreat* or exploit* or fraud* or assault* or crime* or violen* or bully*or ntimidate* or aggressi* or coerc* or extort* or stigmati* or ostraci*).ti,ab. (658570)

7 fraud/ or homicide/ or sex offenses/ or rape/ or theft/ or violence/ or domestic violence/ or elder abuse/ (81700)

8 5 or 6 or 7 (706738)

9 exp Homes for the Aged/ or exp Nursing homes/ or exp Residential Facilities/ or exp Long-Term Care/ or aged specific care.mp. (81846)

10 (Mandatory report* or recogni* or assess* or report* or geriatric assessment or risk assessment).mp. (10672246)

11 (geriatric nursing or nursing staff).mp. (94271)

12 (nursing and (staff or service* or employee* or worker* or professional*)).mp. (258982)

13 11 or 12 (267977)

14 4 and 8 (174707)

15 9 and 14 (1813)

16 13 and 15 (774)

17 10 and 16 (450)
