# Supplementary material for: Global approaches to older abuse research in institutional care settings: A systematic review
Source: PLoS One. 2025 Mar 10;20(3):e0290482. doi: 10.1371/journal.pone.0290482 (PMC11892848; doi:10.1371/journal.pone.0290482)
Supplement: S1 Table — (DOCX) [file pone.0290482.s006.docx]

# **S1** **Table: Study eligibility using PICOTS framework.**

| **Eligibility criterion (PICOTS)** | **Inclusion Criteria** | **Exclusion Criteria** |
| --- | --- | --- |
| **Population (P)** | ***Age:*** Older adults [≥65] and good cognitive function | **Age:** Adults <65 or cognitively impaired |
| **Intervention (I)/Exposure** | ***Type of abuse:*** physical, psychological, financial, sexual abuse, neglect [WHO (2022)] and other abuse  ***Direction of abuse:*** staff-to-resident  ***Reported as*** observed or committed abuse  ***Reported by*** staff, residents, family/relatives or community | ***Type of abuse:*** No methods measuring physical, psychological, financial, sexual abuse, neglect [WHO (2022)] and other abuse  D***irection of abuse****:* resident-to-resident abuse or resident-to-staff abuse |
| **Comparator/Control (C)** | With or without a comparative sample |  |
| **Outcome (O)** | Any incidence or prevalence data, definitions of abuse, methodologies, and incident rate findings | No prevalence or incident data reporting on older adult abuse and/or intervention studies (examining risk factors associated with abuse with no incident or prevalence |
| **Study design and type of publication** | Quantitative and peer-reviewed published papers | Qualitative studies, case studies, case series, conference proceedings, discussion or opinion pieces, meta-analyses, methodological papers, non-published papers, review papers, systematic reviews, theoretical papers, book chapters, case reports, gray literature, and reports |
| **Timing (T)** | No specific timing. |  |
| **Setting (S)** | ***Institution:*** residing in long-term aged care institutional settings [assisted, independent or extended living facilities or care units, and residential or a nursing home] | ***Institution:*** residing in institutional care, short-term care facilities: rehabilitation facilities, palliative care facilities or ***Cognitive function:*** memory care facilities *(dementia care facilities)* |
| **Language** | English | Non-English written articles |
