# Supplementary material for: Global approaches to older abuse research in institutional care settings: A systematic review
Source: PLoS One. 2025 Mar 10;20(3):e0290482. doi: 10.1371/journal.pone.0290482 (PMC11892848; doi:10.1371/journal.pone.0290482)
Supplement: S5 File — (DOCX) [file pone.0290482.s008.docx]

# **S4 File. Data extraction template variables information** eligible studies

**Details of study**

1. Author names
2. Year of Publication
3. Country
4. Continent
5. Title of Study
6. Study Timeframe

**Design, Recruitment and Study Characteristics**

1. What is the study design?
2. How did the study define long-term institutions?
3. Record the study population - Institutional Facilities (both numerator/denominator) [%]
4. Is the response rate recorded? (Y/N)
5. What is the response rate? (%)
6. What is the study’s definition of staff?
7. What is the study population – staff (numerator/denominator) [%]
8. Did the study collect abuse by staff to residents, as reported by staff? (Y/N)
9. Number of females – staff (n, %)
10. What is the mean age of staff in the study [Mean Age (sd)]?
11. What is the reported age range of staff?
12. Record the type of occupation of staff involved in this study.
13. Record the years of employment of staff.
14. What is the study’s definition of resident?
15. What is the study population – resident (numerator/denominator) [%]
16. Did the study collect abuse by staff to residents, as reported by residents (Y/N)?
17. Number of females – resident (n, %)
18. What is the mean age of residents in the study [Mean Age (sd)]?
19. What is the reported age range of residents?
20. What is the comorbidity or Dependency rate (n) – resident
21. What is the mean year of residency – resident
22. What is the study’s definition of a community member?
23. What is the study population – community member (numerator/denominator) [%]
24. Did the study collect abuse by staff to residents, as reported by the community (Y/N)?
25. Number of females – community (n, %)
26. What is the mean age of community members in the study [Mean Age (sd)]?
27. What is the mean year of residency – community member?

**Methodology**

1. Report by [Staff (S), Staff & Resident (S&R), Resident (R), Community member (family, relative) (REL) or registry (ALL)]
2. Description of method of collecting abuse data -Questionnaire, Survey (Face to Face, Online, Mail).
3. Consent by Institute (Y/N or Not reported [NR])
4. Reported Experienced and/or Observed abuse by staff to resident (E or O, E&O)
5. Experienced/Observed abuse question- name
6. Self-Constructed (SC) or Standard Question(s) (SQ)
7. Questionnaire abuse derived from – citation
8. Questionnaire – Number of items
9. Questionnaire – Tested (Y/N)
10. Questionnaire – Reliability Tested – findings
11. Questionnaire – Validity Tested – findings
12. Questionnaire – Reliability – Citation
13. Questionnaire – Validity – Citation
14. Recall Period (months)
15. Frequency of Abuse

**Type of Abuse**

1. Types of abuse [Overall, Physical (P), Psychological (Psych), Financial (F), Sexual (S), Neglect (N) or Other (O)]
2. Number of reported types of abuse (1 – 5 [WHO definition) or more)
3. Measured overall abuse (Y/N)
4. **Overall abuse question**
5. Overall abuse incident (E/O) (Reported by S, S&R, REL, ALL) *
6. **Physical abuse (Y/N)**
7. Physical abuse question
8. Physical abuse number of items
9. Physical abuse incident (E/O)
10. **Psychological abuse (Y/N)**
11. Psychological abuse question
12. Psychological abuse number of items
13. Psychological abuse incident (E/O) (Reported by S, S&R, REL, ALL) *
14. **Financial abuse (Y/N)**
15. Financial abuse question
16. Financial abuse number of items
17. Financial abuse incident (E/O) (Reported by S, S&R, REL, ALL) *
18. **Sexual abuse (Y/N)**
19. Sexual abuse Question
20. Sexual abuse number of items
21. Sexual abuse incident (E/O) (Reported by S, S&R, REL, ALL) *
22. **Neglect abuse (Y/N)**
23. Neglect abuse question
24. Neglect abuse number of items
25. Neglect abuse incident (E/O) (Reported by S, S&R, REL, ALL) *
26. **Other abuse (Y/N)**
27. Other abuse Question
28. Other abuse number of items
29. Other abuse incident (E/O) (Reported by S, S&R, REL, ALL) *

*ALL= public, S= Staff, REL=Relative, S&R=Staff and Relative
