# Supplementary material for: Global approaches to older abuse research in institutional care settings: A systematic review
Source: PLoS One. 2025 Mar 10;20(3):e0290482. doi: 10.1371/journal.pone.0290482 (PMC11892848; doi:10.1371/journal.pone.0290482)
Supplement: S6 File — (DOCX) [file pone.0290482.s009.docx]

# S5 File. Methodological quality criteria list

1. Was the target population defined clearly through inclusion and exclusion criteria? (s)

(1) does the survey design yield a sample of respondent’s representative of a defined target population? Is the target population defined clearly?

2. Was probability sampling used to identify potential respondents (or the whole population approached(s)?

Was probability sampling used to identify potential respondents?

3. Did characteristics of respondents match the target population, i.e.: was the response rate ≥80% with appropriate analysis included comparing responders and non-responders?

Do the characteristics of respondents match the target population?

4. Were data collection methods standardised?

5. Was the abuse measure valid?

6. Was the abuse measure reliable?

7. Were features of sampling design accounted for in the analysis, through appropriate weighting of the data, or the whole population approached? (CIs reported?)

8. Do the reports include confidence intervals for statistical estimates? (Were the estimates of prevalence or incidence given with confidence intervals?)
