# Supplementary material for: Global approaches to older abuse research in institutional care settings: A systematic review
Source: PLoS One. 2025 Mar 10;20(3):e0290482. doi: 10.1371/journal.pone.0290482 (PMC11892848; doi:10.1371/journal.pone.0290482)
Supplement: S2 Table — (DOCX) [file pone.0290482.s010.docx]

# **S2 Table. Aged Care Abuse Research Checklist (ACARC)**

| **Element** | **Key questions** |
| --- | --- |
| Study design | 1) Does the study recruit a representative cross-sectional cohort of participants using a random selection of nursing homes [37,44] or via a professional registration list? [37] |
|  | 2) Does the study include sub-representative groups within institutionalised care settings, from impaired to non-cognitive functioning residents among cognitive functioning residents? [47] |
| Methodology | 3) Does the study conduct data collection discretely with the use of an independent researcher? [26, 44] |
|  | 4) Does the study utilise a standardised validated unmodified definition and measurement tools on abuse of older adults? [65] |
|  | 5) Does the study collect ‘self-reported’*,* observed, committed or experienced forms of abuse from all stakeholders, including staff, residents, relatives and community members? [48] |
|  | 6) Does the study incorporate the collection of signs and symptoms of abuse by using independent professional forensic physicians, other medical staff or an independent researcher in a timely manner? [44, 50, 45, 41, 42] |
|  | 7) Does the study collect data within an agreed recall period - ideally between three to twelve months? [66] |
|  | 8) Does the study collect the incidence of abuse:  a) to measure immediate signs and reports of suspected within a timely manner; and,  b) in-depth to verify incidents of abuse and to investigate the complexities attached to these abuse acts [39, 40, 43, 48] to minimise recall bias. [50, 45, 41, 42] |
| Results | 9) Does the study report on age and sex-specific estimates with confidence intervals to allow study comparisons? [20] |
|  | 10) Does the study present data on all study participants' stakeholders’ characteristics, as risk factors studies have shown these variables are determinates of abuse? [8] |
| Publication | 11) Does the study publish methods and results with transparency - by providing the full questionnaire and additional study results using publisher appendices to improve the study’s validity and reliability? |
