# Supplementary material for: Global approaches to older abuse research in institutional care settings: A systematic review
Source: PLoS One. 2025 Mar 10;20(3):e0290482. doi: 10.1371/journal.pone.0290482 (PMC11892848; doi:10.1371/journal.pone.0290482)
Supplement: S3 Table — (DOCX) [file pone.0290482.s011.docx]

## S3 Table. Definitions of Types of Abuse

## Physical Definition

| **Author, Year & Country** | **Reported by** | **Questionnaire** | | **Abuse Definition & Abuse Description** |
| --- | --- | --- | --- | --- |
| **Ben Natan M et al.**  **2010, IL [26]** | S | Daly & Jogerst (2005), Iowa Dependent Adult Abuse Nursing Home Questionnaire | | *Physical violence -* No description given |
| **Blumenfeld Arens O et al. 2017, SW [47]** | S | Malmedal et al. (2009) See Malmedal | | *Physical abuse 3 items:* Single item measurement: pushing, hitting & kicking a resident |
| **Botngård A et al. 2020 NO [48]** | S | Castle (2012) modified, Verbal: Clarke & Pierson (1999) *Verbal classified as psychological, Psych: Lachs & Pillemer (2004), Caregiver: Federal Register (1991), Medication: Chambers (1999), Material exploitation: Rabiner et al. (2006), Sexual: The National Center on Elder Abuse (1998), Sexual: Malmedal et al. (2009) | | *Acts of physical abuse: 7 items (reported 10):* pushing, grabbing, or pinching a resident, pulling hair or kicking a resident, hurting a resident on purpose, throwing things at a resident, hitting a resident, bullying a resident, behaving aggressively towards a resident, not giving needed medication on purpose to a resident, giving more medication than needed on purpose to a resident & deliberately delaying giving medication(s) to a resident |
| **Buzgova R & Ivanová K, 2011, CR [49]** | R & S | Buzgova & Ivanová et al. (2009) & WHO (2002) | | *Physical abuse: 9 items:* pushing or violent grabbing, attempted slapping, slapping, washing in cold water, excessive use of physical restraints, denial of food or privileges as punishment, throwing an object at a client, hitting a client with an object & kicking |
| **Castle N., 2012, USA [37]** | S | Verbal: Clarke & Pierson (1999), Psych: Lachs & Pillemer (2004), Caregiver: Federal Register (1991), Medication: Chambers (1999), Material exploitation: Rabiner et al. (2006), Sexual: The National Center on Elder Abuse (1998) | | *Physical abuse* defined as acts done with the intention of causing physical pain or injury” (Lachs & Pillemer, 2004, p. 1264). *7 items (reported 5):* pushing, grabbing, or pinching, pulling hair or kicking, other physical violence, deliberately hurting resident & hitting or throwing things at resident |
| **Castle N & Beach S. 2013 USA [38]** | S | Physical developed by Lachs & Pillemer (2004), Verbal developed by Clarke & Pierson (1999), Psych developed by Lachs & Pillemer (2004), Caregiver developed by Federal Register (1991), Medication developed by Chambers (1999), Material exploitation developed from Rabiner et al. (2006), Sexual developed from The National Center on Elder Abuse (1998) | | Physical abuse defined as acts done to cause physical pain or injury” (Lachs & Pillemer, 2004, p. 1264). 7 items (reported 5) 1. pushing, grabbing, or pinching, 2. pulling hair or kicking, 3. other physical violence, 4. deliberately hurt resident & 5. hit or throw things at a resident. |
| **Cohen M et al 2010 IL [44]** | R | Signs of Abuse Inventory and the Expanded Indicators of Abuse Questionnaire questionnaires (Cohen 2006, 2007), Kottwitz & Bowling (2003) & Wan, Tseng and Chen (2007) | | *Disclosure of maltreatment/abuse – physical abuse, Disclosure of episodes of physical abuse.6 Examples:* pushing, hitting, pinching, applying restraint, holding tightly, threatening. The Signs of Abuse Inventory (Cohen et al. 2006, 2007), signs of physical and sexual abuse, financial exploitation and neglect to ‘4’ ‘extreme’. Signs of abuse: for each item on a scale from ‘0’ to ‘not at all’ *7-items:* unexplained injuries or unsatisfactory explanations for their injuries; internal haemorrhage, new and old scars, scars on upper parts of both arms, burns on parts of the body not usually subject to burns, unusual burn, shape of burn (matching certain objects such as  cigarette or flatiron) & dipping burns. |
| **Frazão S L et al. 2015 PT [50]** | ALL | Forensic medical reports (FMR) | | *Medical observations & victim complaints: Physical abuse:* Examples: grasping, pushing and punching, slapping and aggression with scissors. Forensic medical findings are suggestive of physical abuse. Alleged victim of physical abuse in an institutional setting by a caregiver |
| **Friedman L et al. 2017 USA [39]** | ALL | Clinical signs of neglect [CSNS] Items: 0 – 60 items  (highest value: 30) | | NR |
| **Gil AP & Capelas ML**  **2022 PT [51]** | S | Drennan et al. (2012)., Items: 31, Abuse: 5 types, 31 types, last 12 months | | Physical abuse: closing in a room as punishment, tightening very strongly, physical aggression (spanking, pushing, throwing an object, tying the older person to a chair to not have so much work (wrists, belly), using other restraining strategies to not give so much work, using medication to keep the person calmer and to have less work |
| **Griffore RJ et al. 2009, USA [21]** | RT | Michigan Survey of Households with Family Members, Receiving Long-Term Care Services (MLTCS) | | *Physical mistreatment*  *10 items:* striking, hitting, beating, pushing, shoving  Shaking, slapping, kicking, pinching, burning |
| **Habjanič A & Lahe D 2012 SI [52]** | R | Develop from examples primarily from: The National Center on Elder Abuse (1998), Isola et al. (2008), Garre-Olmo et al. (2009) & Malmedal et al. (2009) | | *Physical abuse*  *4 items* slapping, hit or kicked, having visible bruising on your body  being hurt by various items & causing burns to you |
| **Malmedal W et al. 2009 NO [53]** | S | Based on several clinical research studies Saveman BI et al. (1999), Goergen (2001) & Pillemer & Bachman-Prehn (1991) | | *Acts of Inadequate car: acts of physical character*  *5 items* held a resident hard, gave medication when one should not have done it, restrained/held back a resident, pressed the nose in order to force the resident to open his or her mouth, tied down a resident |
| **McCool JJ et al. 2009 USA [43]** | S | Carolyn Lea Clark-Daniels, R. Steven Daniels & Lorin A. Baumhover (1989) Physicians' and Nurses' Responses to Abuse of the Elderly, Oswalkd JHogerst Daily Iowa Journal of Elder Abuse & Neglect, “Nursing Home Questionnaire” | | Physical abuse: Nursing and Administrative Staff who have seen (self-determined definition of physical abuse) [consisted of 31 statements on the definition of abuse, the understanding of mandatory reporting requirements, and willingness to report. Respondents were asked to evaluate these statements on a five-point Likert-type scale ranging from "definitely not true" to "definitely true. ''5 questions: Tell me about the kinds of elder abuse that might be encountered in the nursing home setting” [self-defining -physical] |
| **Moore S 2016 UK [32]** | S | Primary type of recorded abuse was also requested for each case using the classifications within “No Secrets: Guidance on developing and implementing multi-agency policies and procedures to protect vulnerable adults from abuse” (Department of Health, 2000) (UK). | | NR |
| **Moore S. 2020 UK [33]** | S | Data gathering instrument | | NR |
| **Neuberg M et al. 2017 HRV [54]** | S | Drennan J, Lafferty A, Treacy MP, Fealy G, Phelan A, Lyons I, Hall P. Older People in Residential Care Settings: Results from a National Survey of Staff-Resident Interactions and Conflicts. NCPOP: University College Dublin, 2012 [displayed 19 April 2017]. Available at <http://www.rte.ie/> documents/news/elderlyreport.pdf  Part 5: Staff interactions and conflicts with residents, including reports of neglect and abuse. | | 6 items: restrain a resident beyond what was needed at the time, push, grab, shove or pinch a resident, throw something at a resident., slap or hit a resident, kick a resident or hit with a fist., hit or try to hit a resident with an object. |
| **Phillips LR & Ziminski C 2012 USA [40]** | ALL | Review and copy all narrative reports for complaint investigations in public facility files. Types of neglect and neglect-related outcomes were identified from the complaint narratives using manifest content analysis Categorised into Inappropriate care practices: Failure to meet scheduled and unscheduled needs, to provide safety and to notify appropriate persons in response to an emergency or accident, establish and maintain a written care plan, to correctly and safely administer appropriate medications, to provide appropriate nutrition and hydration and/or inappropriate admittance of a resident who does not meet ALF criteria. | NR | |
| **Smith D et al. 2019 AUS [45] (Women Only)** | ALL | Forensic medical reports (FMR) | NR | |
| **Smith DE et al. 2022 AUS [46]** | ALL | Thirteen survey items were analysed: Perceived barriers to reporting Unwanted Sexual behaviour in residential aged care services. | NR | |
| **Teaster P B et al. 2007**  **USA [42] (Men Only)** | ALL | Adult Protective Services (APS) and other regulatory entities from five states & used SASU. | NR | |
| **Teaster P B et al.**  **2015 USA [41] (Women Only)** | ALL | Adult Protective Services (APS) and other regulatory entities from five states & used SASU. | NR | |

## Psychological Definition

| **Ben Natan M et al. 2010 IL [26]** | S | Daly & Jogerst (2005) &Iowa Dependent Adult Abuse Nursing Home Questionnaire | | Mental abuse: No description was given | |
| --- | --- | --- | --- | --- | --- |
| **Blumenfeld Arens O et al., 2017 SW [47]** | S | Malmedal et al. (2009) | | *Emotional Abuse*  *3 items:* scold or yell at a resident, talk disrespectfully to a resident, make fun of a resident in front of others | |
| **Botngård A et al. 2020 NO [48]** | S | Castle (2012) modified, Verbal: Clarke & Pierson (1999), Psych: Lachs & Pillemer (2004), Caregiver: Federal Register (1991), Medication: Chambers (1999), Material exploitation: Rabiner et al. (2006), Sexual: The National Center on Elder Abuse (1998), Sexual: Malmedal et al.(2009) verbal classified as psychological | | *Psychological abuse (3 items) and verbal abuse (5 items)* Psychological abuse: making threatening remarks to a resident, making critical remarks to a resident, threatening to stop taking care of a resident. Verbal abuse: yelling at a resident, making nasty remarks to a resident, swearing at a resident, making humiliating remarks to a resident & arguing with a resident | |
| **Buzgova R & Ivanova K. 2011 CR [49]** | R & S | Buzgova & Ivanová et al.et al.(2009) & WHO (2002) | | *Psychological/emotional abuse*  *14 items:* shouting, infantilizing, intentionally ignoring, intentionally switching off signalling devices, harsh swearing, intentionally dissatisfying clients’ wishes, movement restrictions or bans, verbal offences threats harsh commands intentional isolation, intentional humiliation, limiting the ability to make decisions or participate in care, gross violation of privacy | |
| **Castle N. 2012 USA [37]** | S | | Verbal: Clarke & Pierson (1999), Psych: Lachs & Pillemer (2004), Caregiver: Federal Register (1991), Medication: Chambers (1999), Material exploitation: Rabiner et al. (2006), Sexual: The National Center on Elder Abuse (1998) | | *Psychological abuse* is defined as “acts done with the intention of causing emotional pain or injury” (Lachs & Pillemer, 2004, p. 1264). *4 items* specifically ask questions on intimidation, aggressive behaviour, threatening remarks, and critical remarks. *5 items (verbal)* yelling, insulting remarks, cursing, argumentative with residents, humiliating remarks. |
| **Castle N & Beach S. 2013 USA [38]** | S | | Physical developed by Lachs & Pillemer (2004), Verbal developed by Clarke & Pierson (1999), Psych developed by Lachs & Pillemer (2004), Caregiver developed by Federal Register (1991), Medication developed by Chambers (1999), Material exploitation developed from Rabiner et al. (2006), Sexual developed from The National Center on Elder Abuse (1998) | | Psychological abuse is defined as “acts done with the intention of causing emotional pain or injury” (Lachs & Pillemer, 2004, p. 1264). 4 items specifically ask questions on intimidation, aggressive behaviour, threatening remarks, and critical remarks. 5 items (verbal) yelling, insulting remarks, cursing, argumentative with residents, humiliating remarks. |
| **Cohen M et al. 2010**  **IL [44]** | R | | Signs of Abuse Inventory and the Expanded Indicators of Abuse Questionnaire questionnaires (Cohen 2006, 2007) Kottwitz & Bowling (2003) &Wan, Tseng and Chen (2007) | | *Disclosure of maltreatment/abuse – psychological Abuse. 5 ‘examples:* insults, ridicule, shouting, swearing, deliberately angering the respondent. ‘Signs of psychological abuse were not assessed, as they are almost impossible to detect without direct observation or personal reports. |
| **Frazão S L et al. 2015 PT [50]** | ALL | | Forensic medical reports (FMR) | | NR |
| **Friedman L et al. 2017 USA [39]** | ALL | | Clinical signs of neglect [CSNS] Items: 0 – 60 items  (highest value: 30) | | NR |
| **Gil AP & Capela ML 2022 PT [51]** | S | | Drennan et al. (2012), Items: 31 Abuse: 5 types, 31 types, last 12 months | | Psychological abuse  12 items: shouting at a resident, intentionally ignoring a resident when they call, rejecting an older person, giving nicknames in a pejorative way (to hurt), humiliating to make them feel ashamed, punishing (not taking to the living room, or to the garden), not respecting privacy, provoking the older person in a deliberate manner until causing anger, verbally threatening the older person verbally insulting (calling names), punishing by refusing access to a meal (food, drink) & spreading rumours about residents |
| **Griffore R J et al. 2009 USA [21]** | RT | | Michigan Survey of Households with Family Members Receiving Long-Term Care Services (MLTCS) | | *Emotional or psychological mistreatment:* treated disrespectful, disallowing contact with family, or giving the silent treatment. *Verbal mistreatment:* yelling, cursing or swearing, insult threats, intimidation, humiliation or harassment |
| **Habjanič A & Lahe D 2012 SI [52]** | R | | Develop from examples primarily from: The National Center on Elder Abuse (1998), Isola et al. (2008), Garre-Olmo et al. (2009), Malmedal et al. (2009) | | NR |
| **Malmedal W et al. 2009 NO [53]** | S | | Based on several clinical research studies, Saveman BI et al. (1999), Goergen (2001), Pillemer & Bachman-Prehn (1991) | | *Emotional abuse: 7 items* scolded a resident, entered a resident’s room without knocking, threatened a resident with punishment, made fun of a resident in front of others talked disrespectfully to a resident, prohibited a resident from using the alarm, used diapers to prevent toilet visits |
| **McCool JJ et al. 2009 USA [43]** | S | | Carolyn Lea Clark-Daniels, R. Steven Daniels & Lorin A. Baumhover (1989) Physicians' and Nurses' Responses to Abuse of the Elderly. Oswalkd JHogerst Daily Iowa Journal of Elder Abuse & Neglect, " Nursing Home Questionnaire” | | Emotional abuse: Nursing and Administrative Staff who have seen (self-determined definition of physical abuse) [consisted of 31 statements on the definition of abuse, the understanding of mandatory reporting requirements, and willingness to report. Respondents were asked to evaluate these statements on a five-point Likert-type scale ranging from "definitely not true" to "definitely true. ''5 questions: Tell me about the kinds of elder abuse that might be encountered in the nursing home setting” [self-defining - physical] |
| **Moore S 2016 UK [32]** | S | | Primary type of recorded abuse was also requested for each case using the classifications: within “No Secrets: Guidance on developing and implementing multi-agency policies and procedures to protect vulnerable adults from abuse” (Department of Health, 2000) (UK). | | Re-categorisation of “psychological abuse” to “emotional abuse. 13 items: emotional abuse, threats of harm, abandonment, deprivation of contact, humiliation, blaming, controlling, intimidation, coercion, harassment, verbal abuse, isolation, withdrawal from services or supportive networks |
| **Moore S 2020 UK [33]** | S | | Data gathering instrument | | NR |
| **Neuberg M et al. 2017 HRV [54]** | S | | Drennan J, Lafferty A, Treacy MP, Fealy G, Phelan A, Lyons I, Hall P. Older People in Residential Care Settings: Results from a National Survey of Staff-Resident Interactions and Conflicts. NCPOP: University College Dublin, 2012. Part 5: Staff interactions and conflicts with residents, including reports of neglect and abuse | | 6 items: isolate a resident beyond what was needed to control him or her, insult or swear at a resident, shout at a resident in anger, deny a resident food or privileges as part of a punishment, threaten to hit or throw something at a resident, hit or try to hit a resident with an object. |
| **Phillips LR & Ziminski C 2012 USA [40]** | S | | Review and copy all narrative reports for complaint investigations in public facility files. Types of neglect and neglect-related outcomes were identified from the complaint narratives using manifest content analysis Categorised into Inappropriate care practices: Failure to meet scheduled and unscheduled needs, to provide safety and to notify appropriate persons in response to an emergency or accident, establish and maintain a written care plan, to correctly and safely administer appropriate medications, to provide appropriate nutrition and hydration and/or inappropriate admittance of a resident who does not meet ALF criteria. | | NR |
| **Smith D et al. 2019 AUS [45] (Women Only)** | ALL | | Forensic medical reports (FMR) | | NR |
| **Smith DE et al. 2022**  **AUS [46]** | ALL | | Thirteen survey items were analysed: Perceived barriers to reporting Unwanted Sexual behaviour in residential aged care services. | | NR |
| **Teaster P B et al 2007 USA [42] (Men Only)** | ALL | | Adult Protective Services (APS) and other regulatory entities from five states & used SASU. | | NR |
| **Teaster P B et al. 2015 USA [41] (Women Only)** | ALL | | Adult Protective Services (APS) and other regulatory entities from five states & used SASU. | | NR |
| ***Financial Definition*** |  | |  | |  |
| **Ben Natan M et al 2010 IL [26]** | S | | Daly & Jogerst (2005) & Iowa Dependent Adult Abuse Nursing Home Questionnaire | | Financial exploitation  No description given |
| **Blumenfeld Arens O et al 2017 SW [47]** | S | | Malmedal et al. (2009) | | NR |
| **Botngård A et al 2020 NO [48]** | S | | Castle (2012) modified Verbal: Clarke & Pierson (1999) Psych: Lachs & Pillemer (2004) Caregiver: Federal Register (1991) Medication: Chambers (1999) Material exploitation: Rabiner et al. (2006), Sexual: The National Center on Elder Abuse (1998), Sexual: Malmedal et al. (2009), verbal classified as psychological. | | *Material/Financial exploitation: 4 items* stealing money from a resident, stealing things from a resident, signing documents without permission from a resident, destroying things that belong to a resident without permission |
| **Buzgova R & Ivanová K 2011 CR [49]** | R & S | | Buzgova & Ivanová et al. (2009) & WHO (2002) | | NR |
| **Castle N 2012 USA [37]** | S | | Physical developed from Lachs & Pillemer (2004), verbal developed from Clarke & Pierson (1999), psych developed from Lachs & Pillemer (2004), caregiver developed from federal register (1991), medication developed from chambers (1999), material exploitation developed from Rabiner et al. (2006), sexual developed from the National Center on Elder Abuse (1998). | | *Material exploitation - “*the improper use of an older person’s assets” (Rabiner, O’Keeffe, & Brown, 2006, p. 51).*5 items* taking possessions, taking assets, destroyed belongings, sign checks without permission, tamper with savings. |
| **Castle N & Beach 2011 USA [38]** | S | | Physical developed by Lachs & Pillemer (2004), Verbal developed by Clarke & Pierson (1999), Psych developed by Lachs & Pillemer (2004), Caregiver developed by Federal Register (1991), Medication developed by Chambers (1999), Material exploitation developed from Rabiner et al. (2006), Sexual developed from The National Center on Elder Abuse (1998) | | *Material exploitation - “the improper use of an older person’s assets” (Rabiner, O’Keeffe, & Brown, 2006, p. 51).* 5 items taking possessions, taking assets, sign checks without permission, tamper with savings, destroy belongings. |
| **Cohen M et al 2010 IL [44]** | R | | Signs of Abuse Inventory and the Expanded Indicators of Abuse Questionnaire questionnaires (Cohen 2006, 2007) Kottwitz & Bowling (2003) &Wan, Tseng and Chen (2007) | | *Financial exploitation: Signs of Neglect:* The Signs of Abuse Inventory (Cohen et al. 2006, 2007), signs of physical and sexual abuse, financial exploitation and neglect to ‘4’ ‘extreme’. Signs of abuse: for each item on a scale from ‘0’ ‘not at all’ *7 items (reported 5):* Indirect evidence of exploitation: cannot satisfy basic needs even though sufficient financial resources exist, lack of orientation regarding bank account [if cognitively capable]), direct evidence of exploitation, enforced transfer of property, signing documents, giving money |
| **Frazão S L et al 2015 PT [50]** | ALL | | Forensic medical reports (FMR) | | NR |
| **Friedman L et al 2017 USA [39]** | ALL | | Clinical signs of neglect [CSNS], Items: 0 – 60 items  (highest value: 30) | | NR |
| **Gil AP & Capelas ML 2022 PT [51]** | S | | Drennan et al. (2012), Items: 31, Abuse: 5 types, 31 types, last 12 months | | 1 item: misappropriation of property (money, jewellery, clothing or other personal property) |
| **Griffore RJ et al 2009 USA [21]** | RT | | Michigan Survey of Households with Family Members & Receiving Long-Term Care Services (MLTCS) | | *Material exploitation: 4 items misuse* of a person’s funds, property or assets, theft of money or possessions, coercion or deception into signing documents or gifting possessions |
| **Habjanič A & Lahe D 2012, SI [52]** | R | | Develop from examples primarily from: The National Center on Elder Abuse (1998), Isola et al. (2008), Garre-Olmo et al. (2009) & Malmedal et al. (2009) | | *Financial abuse: 6 items* denial of the right to spend your own money, being unnecessarily charged money, taking possession of your pension without letting you know, you are forced to sign documents against your will, forging your signature, demands that a will be changed in someone favour |
| **Malmedal W et al. 2009 NO [53]** | S | | Based on several clinical research studies Saveman BI et al. (1999), Goergen (2001), & Pillemer & Bachman-Prehn (1991) | | *Acts of inadequate care character: 1 item: t*aken money or valuables from a resident |
| **McCool JJ et al. 2009 USA [43]** | S | | Carolyn Lea Clark-Daniels, R. Steven Daniels & Lorin A. Baumhover (1989) Physicians' and Nurses' Responses to Abuse of the Elderly, Oswalkd JHogerst Daily Iowa Journal of Elder Abuse & Neglect, " Nursing Home Questionnaire” | | Financial Exploitation: Nursing and Administrative Staff who have seen (self-determined definition of physical abuse) [consisted of 31 statements on the definition of abuse, the understanding of mandatory reporting requirements, and willingness to report. Respondents were asked to evaluate these statements on a five-point Likert-type scale ranging from "definitely not true" to "definitely true. '' 5 questions: Tell me about the kinds of elder abuse that might be encountered in the nursing home setting” [self-defining - physical] |
| **Moore S 2016 UK [32]** | S | | Primary type of recorded abuse was also requested for each case using the classifications within “No Secrets: Guidance on developing and implementing multi-agency policies and procedures to protect vulnerable adults from abuse” (Department of Health, 2000).(UK) | | 9 items: theft, fraud, exploitation, pressure in connection with wills, property transactions, inheritance transactions, financial transactions, the misuse or misappropriation of property, possessions or benefits |
| **Moore S 2020 UK [33]** | S | | Data gathering instrument | | NR |
| **Neuberg M et al. 2017 HRV [54]** | S | | Drennan J, Lafferty A, Treacy MP, Fealy G, Phelan A, Lyons I, Hall P. Older People in Residential Care Settings: Results from a National Survey of Staff-Resident Interactions and Conflicts. NCPOP: University College Dublin, 2012, Part 5: Staff interactions and conflicts with residents, including reports of neglect and abuse. | | *1 item: t*ake jewellery, money, clothing or something else from a resident or resident’s room. |
| **Phillips LR & Ziminski C 2012 USA [40]** | ALL | | Review and copy all narrative reports for complaint investigations in public facility files. Types of neglect and neglect-related outcomes were identified from the complaint narratives using manifest content analysis | | NR |
| **Smith D et al. 2019 AUS [45] (Women Only)** | ALL | | Forensic medical reports (FMR) | | NR |
| **Smith DE et al. 2022 AUS [46]** | ALL | | Thirteen survey items were analysed: Perceived barriers to reporting Unwanted Sexual behaviour in residential aged care services | | NR |
| **Teaster P B et al.**  **2007 USA [42] (Men Only)** | ALL | | Adult Protective Services (APS) and other regulatory entities from five states & used SASU | | NR |
| **Teaster P B et al.**  **2015 USA [41] (Women Only)** | ALL | | Adult Protective Services (APS) and other regulatory entities from five states & used SASU | | NR |
| ***Sexual Definition*** |  | |  | |  |
| **Ben Natan M et al. 2010 IL [26]** | S | | Daly & Jogerst (2005) Iowa Dependent Adult Abuse Nursing Home Questionnaire | | Sexual violence  No description given |
| **Blumenfeld Arens O et al. 2017 SW [47]** | S | | Malmedal et al. (2009) | | NR |
| **Botngård A et al. 2020 NO [48]** | S | | Castle (2012) modified Verbal: Clarke & Pierson (1999) Psych: Lachs & Pillemer (2004) Caregiver: Federal Register (1991) Medication: Chambers (1999) Material exploitation: Rabiner et al. (2006), Sexual: The National Center on Elder Abuse (1998), Sexual: Malmedal et al. (2009), verbal classified as psychological | | *Sexual abuse 4 items (reported 5):* Unwelcome touching of a resident, unwelcome discussion of sexual activity with a resident, exposure of a resident's private-body parts to embarrass them, digital penetration (e.g., finger) of a resident, rape of a resident |
| **Buzgova R & Ivanová K. 2011 CR [49]** | R & S | | Buzgova & Ivanová et al. (2009) & WHO (2002) | | *Sexual Abuse*  NR |
| **Castle N. 2012 USA [37]** | S | | Verbal: Clarke & Pierson (1999), Psych: Lachs & Pillemer (2004), Caregiver: Federal Register (1991), Medication: Chambers (1999), Material exploitation: Rabiner et al. (2006), Sexual: The National Center on Elder Abuse (1998) Material exploitation: Rabiner et al. (2006), Sexual: The National Center on Elder Abuse (1998) | | *Sexual abuse* is defined as “non-consenting sexual contact of any kind” (National Center on Elder Abuse).  *7 items:* unwelcome sexualized kissing, unwelcome fondling, unwelcome discussion of sexual activity, exposure of private body parts to embarrass, oral-genital contact, digital penetration, vaginal rape |
| **Castle N & Beach S. 2013 USA [38]** | S | | Physical developed by Lachs & Pillemer (2004), Verbal developed by Clarke & Pierson (1999), Psych developed by Lachs & Pillemer (2004), Caregiver developed by Federal Register (1991), Medication developed by Chambers (1999), Material exploitation was developed from Rabiner et al. (2006), Sexual developed from The National Center on Elder Abuse (1998) | | Sexual abuse is defined as “non-consenting sexual contact of any kind” (National Center on Elder Abuse).  7 items  unwelcome fondling, unwelcome discussion of sexual activity, exposure of private body parts to embarrass, oral-genital contact, digital penetration, unwelcome sexualized kissing, vaginal rape |
| **Cohen M et al 2010 IL [44]** | R | | Signs of Abuse Inventory and the Expanded Indicators of Abuse Questionnaire questionnaires (Cohen 2006, 2007) Kottwitz & Bowling (2003) &Wan, Tseng and Chen (2007) | | *Disclosure of maltreatment/abuse – sexual abuse.* The Signs of Abuse Inventory (Cohen et al. 2006, 2007), signs of physical and sexual abuse, financial exploitation and neglect to ‘4’ ‘extreme’. Signs of abuse: for each item on a scale from ‘0’ to ‘not at all’ . *4 items:* torn underwear, stained underwear, disease of genitals, infection of genitals |
| **Frazão S L et al. 2015 PT [50]** | ALL | | Forensic medical reports (FMR) | | NR |
| **Friedman L et al. 2017 USA [39]** | ALL | | Clinical signs of neglect [CSNS] Items: 0 – 60 items  (highest value: 30) | | NR |
| **Gil AP & Capelas ML 2022 PT [51]** | S | | Drennan et al. (2012)., Items: 31, Abuse: 5 types, 31 types, last 12 months | | 1 item: Subject the person to contact of a sexual nature without consent. |
| **Griffore RJ et al. 2009 USA [21]** | Relative | | Michigan Survey of Households with Family Members Receiving Long-Term Care Services (MLTCS) | | *Sexual misconduct, reported at least one incident of sexual abuse 4 items:* forced sex, sexual contact without consent, sexual coercion, and unwanted touching. |
| **Habjanič A & Lahe D 2012 SI [52]** | R | | Develop from examples primarily from: The National Center on Elder Abuse (1998), Isola et al. (2008), Garre-Olmo et al. (2009) & Malmedal et al. (2009) Malmedal et al. (2009) | | NR |
| **Malmedal W et al. 2009 NO [53]** | S | | Based on several clinical research studies Saveman BI et al. (1999), Goergen (2001), & Pillemer & Bachman-Prehn (1991)Pillemer & Bachman-Prehn (1991) | | NR |
| **McCool JJ et al. 2009 USA [43]** | S | | Carolyn Lea Clark-Daniels, R. Steven Daniels & Lorin A. Baumhover (1989) Physicians' and Nurses' Responses to Abuse of the Elderly | | Sexual Abuse |
| **Moore S 2016 UK [32]** | S | | Primary type of recorded abuse was also requested for each case using the classifications within “No Secrets: Guidance on developing and implementing multi-agency policies and procedures to protect vulnerable adults from abuse” (Department of Health, 2000).(UK) | | 5 items: rape, sexual assault, sexual acts to which the vulnerable adult has not consented, could not consent and was pressured into consenting. |
| **Moore S 2020 UK [33]** | S | | Data gathering instrument | | NR |
| **Neuberg M et al. 2017 HRV [54]** | S | | Drennan J, Lafferty A, Treacy MP, Fealy G, Phelan A, Lyons I, Hall P. Older People in Residential Care Settings: Results from a National Survey of Staff-Resident Interactions and Conflicts. NCPOP: University College Dublin, 2012. Part 5: Staff interactions and conflicts with residents, including reports of neglect and abuse. | | 1 item  1.       Talk to or touch a resident in a sexually inappropriate way. |
| **Phillips LR & Ziminski C 2012 USA [40]** | ALL | | Review and copy all narrative reports for complaint investigations in public facility files. Types of neglect and neglect-related outcomes were identified from the complaint narratives using manifest content analysis. Categorised into Inappropriate care practices: Failure to meet scheduled and unscheduled needs, to provide safety and to notify appropriate persons in response to an emergency or accident, establish and maintain a written care plan, to correctly and safely administer appropriate medications, to provide appropriate nutrition and hydration and/or inappropriate admittance of a resident who does not meet ALF criteria | | NR |
| **Smith D et al. 2019 AUS [45]**  **(Women Only)** | ALL | | Forensic medical reports (FMR) | | *Alleged incidents of observed or direct sexual abuse Clinical forensic examiner for a forensic medical examination(sexual assault examinations).* Forensic medical examiner responded within 72 hrs of reporting. This includes substantiated cases of allegations of sexual abuse. No description given |
| **Smith DE et al. 2022 AUS [46]** | ALL | | Thirteen survey items were analysed: Perceived barriers to reporting Unwanted Sexual behaviour in residential aged care services | | Awareness of unlawful sexual contact/non-contact between residents (R-R) and staff-resident (S-R) was measured on a 4-point (unlawful: ‘Once a week’-‘Once a decade’) or a 5-point scale (unwelcome: ‘Once a day’-‘ Once a decade’). |
| **Teaster P B et al. 2007 USA [42] (Men Only)** | ALL | | Adult Protective Services (APS) and other regulatory entities from five states & used SASU | | *Sexual Abuse (specific to men)*  Inappropriate sexual behaviour related to sexual interest in victim’s body  Oral genital contact  Digital penetration of anus  Sexual jokes and comments  Anal rape  Exposure to embarrass or humiliate  Showing victim pornography  Fondling |
| **Teaster P B et al. 2015 USA [41] (Women Only)** | ALL | | Adult Protective Services (APS) and other regulatory entities from five states & used SASU | | *Sexual victimization (specific to females). Hands-on offenses:* vaginal rape/attempted vaginal rape, anal rape, digital penetration of vagina or anus, molestation, sexualized kissing, oral genital contact, harmful genital practices, exposure to embarrass or humiliate *Hands-off offenses:* sexual jokes and comments, exhibitionism, inappropriate sexual behaviour related to sexual interest in victim’s body |
| ***Neglect Definition*** |  | |  | |  |
| **Ben Natan M et al.**  **2010**  **IL** | S | | Daly & Jogerst (2005) & Iowa Dependent Adult Abuse Nursing Home Questionnaire | | *Physical neglect and mental neglect:* No description was given |
| **Blumenfeld Arens O et al. 2017 SW [47]** | S | | Malmedal et al. (2009) | | *Neglect: 1 item:* Make a resident wait for required care assistance longer than necessary |
| **Botngård A et al. 2020 NO [48]** | S | | Castle (2012) modified Verbal: Clarke & Pierson (1999) Psych: Lachs & Pillemer (2004) Caregiver: Federal Register (1991) Medication: Chambers (1999) Material exploitation: Rabiner et al. (2006), Sexual: The National Center on Elder Abuse (1998), Sexual: Malmedal et al. (2009), verbal classified as psychological | | *Neglect: 8 items:* not giving food on purpose to a resident, not giving fluid on purpose to a resident, delaying care of a resident, ignoring a resident, not treating a resident’s wounds carefully enough, neglecting oral care of a resident, not changing diapers on a resident, prohibiting a resident from using the alarm |
| **Buzgova R & Ivanová K. 2011 CR [49]** | R & S | | Buzgova & Ivanová et al.(2009) & WHO (2002) | | *Neglect (violation of rights), care neglect:* No description given |
| **Castle N. 2012 USA [37]** | S | | Physical developed from Lachs & Pillemer (2004), verbal developed from Clarke & Pierson (1999), psych developed from Lachs & Pillemer (2004), caregiver developed from federal register (1991), medication developed from chambers (1999), material exploitation developed from Rabiner et al. (2006), sexual developed from the National Center on Elder Abuse (1998). Caregiver was developed by Federal Register (1991) Medication was developed by Chambers (1999) Material exploitation was developed by Rabiner et al. (2006) Sexual developed by The National Center on Elder Abuse (1998) | | NR |
| **Castle N & Beach S. 2013 USA [38]** | S | | Physical developed by Lachs & Pillemer (2004), Verbal developed by Clarke & Pierson (1999), Psych developed by Lachs & Pillemer (2004), Caregiver developed by Federal Register (1991), Medication developed by Chambers (1999), Material exploitation developed from Rabiner et al. (2006), Sexual developed from The National Center on Elder Abuse (1998) | | NR |
| **Cohen M et al. 2010 IL [44]** | R | | Signs of Abuse Inventory and the Expanded Indicators of Abuse Questionnaire questionnaires (Cohen 2006, 2007) Kottwitz & Bowling (2003) &Wan, Tseng and Chen (2007) | | *Neglect of personal basic needs: 7 items:* food or drink not being provided when requested not being taken to bathe, not being cleaned, when necessary, not being taken outdoors, not having bedclothes, changed as needed, and requests for help not being answered, |
| **Frazão S L et al. 2015 PT [50]** | ALL | | Forensic medical reports (FMR) | | *Forensic medical findings suggestive of physical neglect: 2 items: a*ssociated with hygiene neglect were described and medication and medical care neglect were described |
| **Friedman L et al. 2017 USA [39]** | ALL | | Clinical signs of neglect [CSNS], Items: 0 – 60 items (highest value: 30) | | *Clinical Signs Of Neglect Scale (CSNS):* Individual features of the CSNS: *11 items (score):* (1) patients admitted for poisoning as a result of a medication, injuries caused by excessive heat or cold among those affected by conditions that restrict activities of daily living including dementia, disorders of the central nervous system, disorders of the peripheral nervous system, depression, adjustment reaction, osteoarthropathy, and neurological impairments caused by cerebrovascular accident, as well as (2) patients presenting with conditions indicating physical decline that could be the result of neglect including cachexia, failure to thrive, protein-calorie malnutrition, dehydration, pressure ulcers on weight-bearing parts of the body, fecal impaction, complications from poor management of a Foley catheter, colostomy, and enterostomy, muscle or tendon contracture, and aspiration pneumonia |
| **Gil AP & Capelas ML**  **2022 PT [51]** | S | | Drennan et al. (2012).  Items: 31  Abuse: 5 types, 31 types, last 12 months | | 10 items: not changing the position of a bedridden older person, not caring about hygiene (shaving, combing, brushing teeth), not caring about hygiene (not showering), not changing underwear, making an older person wait on purpose, placing diapers without this being necessary to avoid helping to go to the toilet, intentionally making someone wait to go to the toilet. Leaving the older person in bed all day, giving poor information about medication, and disrespecting special diets. |
| **Griffore RJ et al. 2009 USA [21]** | Relative | | Michigan Survey of Households with Family Members Receiving Long-Term Care Services (MLTCS) | | Neglect, 'reported at least one incident of neglect. *8 items:* Failure to rotate provide food, water, shelter, hygiene medicine, comfort, and personal safety. |
| **Habjanič A & Lahe D 2012 SI [52]** | R | | Develop from examples primarily from: The National Center on Elder Abuse (1998), Isola et al. (2008), Garre-Olmo et al. (2009) & Malmedal et al. (2009) | | NR |
| **Malmedal W et al. 2009 NO [53]** | S | | Based on several clinical research studies Saveman BI et al. (1999), Goergen (2001) & Pillemer & Bachman-Prehn (1991)) | | *Acts of negligent: 7 items:* did not wash a resident who needed washing, omitted giving a resident enough food, delayed required care assistance longer than necessary, ignored a resident, inadequate treatment of wounds or injuries, neglected oral care, did not change diapers when needed |
| **McCool JJ et al. 2009 USA [43]** | S | | Carolyn Lea Clark-Daniels, R. Steven Daniels & Lorin A. Baumhover (1989) Physicians' and Nurses' Responses to Abuse of the Elderly Oswalkd JHogerst Daily Iowa Journal of Elder Abuse & Neglect, " Nursing Home Questionnaire” | | Neglect  Nursing and Administrative Staff who have seen (self-determined definition of physical abuse) [consisted of 31 statements on the definition of abuse, the understanding of mandatory reporting requirements, and willingness to report. Respondents were asked to evaluate these statements on a five-point Likert-type scale ranging from "definitely not true" to "definitely true. ''  5 questions: Tell me about the kinds of elder abuse that might be encountered in the nursing home setting” [self-defining - physical] |
| **Moore S 2016 UK [32]** | S | | Primary type of recorded abuse was also requested for each case using the classifications within “No Secrets: Guidance on developing and implementing multi-agency policies and procedures to protect vulnerable adults from abuse” (Department of Health, 2000) (UK) | | Neglect and acts of omission 7 items ignoring medical or physical care needs, failure to provide access to appropriate health, social care, educational services, the withholding of the necessities of life: medication, the withholding of the necessities of life: adequate nutrition, the withholding of the necessities of life: heating. |
| **Moore S 2020 UK [33]** | S | | Data gathering instrument | | NR |
| **Neuberg M et al. 2017 HRV [54]** | S | | Drennan J, Lafferty A, Treacy MP, Fealy G, Phelan A, Lyons  I, Hall P. Older People in Residential Care Settings: Results from a National Survey of Staff-Resident Interactions and  Conflicts. NCPOP: University College Dublin, 2012  [displayed 19 April 2017]. Available at http://www.rte.ie/  documents/news/elderlyreport.pdf  Part 5: Staff interactions and conflicts with  residents, including reports of neglect and abuse. | | 7 items: not change a resident each time they were wet or soiled after an episode of incontinence, ignored a resident when they called, not bring a resident to the toilet when they asked, give a resident too much medication to keep them sedated/quiet, refuse to help a resident with their hygiene needs, refuse to help a resident with their feeding needs, neglect to turn or move a resident to prevent pressure sores. |
| **Phillips LR & Ziminski C 2012 USA [40]** | ALL | | Review and copy all narrative reports for complaint investigations in public facility files. Types of neglect and neglect-related outcomes were identified from the complaint narratives using manifest content analysis | | 1. Three types of neglect were included: Environmental Neglect: an unsafe or unsanitary environment. Medical Neglect: delayed or inappropriate medical treatment, deprivation of health care services outside facility, or medication mismanagement. Personal Neglect: failure to provide goods or services necessary to avoid physical harm, mental anguish, or mental illness. |
| **Smith D et al. 2019**  **AUS [45] (Women Only)** | ALL | | Forensic medical reports (FMR) | | NR |
| **Smith DE et al. 2022 AUS [46]** | ALL | | Thirteen survey items were analysed: Perceived barriers to reporting Unwanted Sexual behaviour in residential aged care services | | NR |
| **Teaster P B et al. 2007 USA [42]**  **(Men Only)** | ALL | | Adult Protective Services (APS) and other regulatory entities from five states & used SASU | | NR |
| **Teaster P B et al. 2015 USA [41] (Women Only)** | ALL | | Adult Protective Services (APS) and other regulatory entities from five states & used SASU | | NR |
| ***Other Definitions*** |  | |  | |  |
| **Ben Natan M et al. 2010 IL [26]** | S | | Daly & Jogerst (2005) & Iowa Dependent Adult Abuse Nursing Home Questionnaire | | NR |
| **Blumenfeld Arens O et al. 2017 SW [47]** | S | | Malmedal et al. (2009) | | NR |
| **Botngård A et al. 2020 NO [48]** | S | | Castle (2012) modified Verbal: Clarke & Pierson (1999) Psych: Lachs & Pillemer (2004) Caregiver: Federal Register (1991) Medication: Chambers (1999) Material exploitation: Rabiner et al. (2006), Sexual: The National Center on Elder Abuse (1998), Sexual: Malmedal et al. (2009), verbal classified as psychological | | *Caregiving abuse*: *2 items:* No descriptions given |
| **Buzgova R & Ivanová K. 2011 CR [49]** | R & S | | Buzgova & Ivanová et al.(2009) &WHO (2002) | | *Violation of rights:* No description given |
| **Castle N 2012, USA [37]** | S | | Verbal: Clarke & Pierson (1999), Psych: Lachs & Pillemer (2004), Caregiver: Federal Register (1991), Medication: Chambers (1999), Material exploitation: Rabiner et al. (2006), Sexual: The National Center on Elder Abuse (1998) Sexual developed from The National Center on Elder Abuse (1998) | | *Medication abuse* is defined as elders “purposely deprived of their correct medication or given inappropriate medication” (Chambers, 1999, p. 80). *3 items:* withholding medications, excessive medications, and access to medications. *Caregiving abuse* is defined as “failure to provide goods and services necessary to avoid physical harm, mental anguish or mental illness” (Federal Register, 1991, p. 48887). *Caregiving abuse is defined as “failure to provide goods and services necessary to avoid physical harm, mental anguish or mental illness” (Federal Register, 1991, p. 48887). 3 items:* threatened to stop taking care of a resident, deliberately withheld food, deliberately withheld water. |
| **Castle N & Beach S., 2013, USA [38]** | S | | Physical developed from Lachs & Pillemer (2004), Verbal developed from Clarke & Pierson (1999), Psych developed from Lachs & Pillemer (2004), Caregiver developed from Federal Register (1991), Medication developed from Chambers (1999), Material exploitation developed from Rabiner et al. (2006), Sexual developed from The National Center on Elder Abuse (1998) | | *Medication abuse is defined as elders “purposely deprived of their correct medication or given inappropriate medication” (Chambers, 1999, p. 80). 3 items:* denied access to medications. given excessive medication, inappropriately delayed giving medication. *Caregiving abuse is defined as “failure to provide goods and services necessary to avoid physical harm, mental anguish or mental illness” (Federal Register, 1991, p. 48887). 3 items:* threatened to stop taking care of a resident, *d*eliberately withheld food, deliberately withheld water. |
| **Cohen M et al., 2010, IL [44]** | R | | Signs of Abuse Inventory and the Expanded Indicators of Abuse Questionnaire questionnaires (Cohen 2006, 2007) Kottwitz & Bowling (2003) &Wan, Tseng and Chen (2007) | | *Disrespectful attitudes:* NR. *6 items:* humiliation behaviour, violation of personal privacy, flouting the requirements of personal modesty, diapering when not necessary, being ignored, giving help impatiently. |
| **Frazão S L et al., 2015, PT [50]** | ALL | | Forensic medical reports (FMR) | | *Caregiving abuse*  *Medical and Medication abuse*  No description given  *Other: combination of one or more types of abuse*: Psychological abuse (threat of physical aggression and death threat) and physical abuse (with a belt), associated with hygiene neglect were described in a case. |
| **Friedman L et al 2017 USA [39]** | ALL | | Clinical signs of neglect [CSNS] Items: 0 – 60 items  (highest value: 30) | | NR |
| **Gil AP & Capelas ML 2022, PT [51]** | S | | Drennan et al. (2012)., Items: 31, Abuse: 5 types, 31 types, last 12 months | | Global (At least 1 of the 24 behaviours observed for the 4 types of abuse and (psychological, physical, financial and neglect). |
| **Griffore R J et al 2009 USA [21]** | Relative | | Michigan Survey of Households with Family Members Receiving Long-Term Care Services (MLTCS) | | *Caretaker abuse: 5 items:* over administration of drugs*, w*ithholding or delaying of drugs*, i*nappropriate use of physical restraints*, u*njustified force feeding & inappropriate toileting practices |
| **Habjanič A & Lahe D 2012, SI [52]** | R | | Develop from examples primarily from: The National Center on Elder Abuse (1998), Isola et al. (2008), Garre-Olmo et al. (2009) & Malmedal et al. (2009) | | NR |
| **Malmedal W et al. 2009 NO [53]** | S | | Based on several clinical research studies, Saveman BI et al. (1999), Goergen (2001), Pillemer & Bachman-Prehn (1991) | | NR |
| **McCool JJ et al. 2009 USA [43]** | S | | Carolyn Lea Clark-Daniels, R. Steven Daniels & Lorin A. Baumhover (1989) Physicians' and Nurses' Responses to Abuse of the Elderly Oswalkd JHogerst Daily Iowa Journal of Elder Abuse & Neglect, "Nursing Home Questionnaire” | | NR |
| **Moore S., 2016 UK [32]** | S | | Primary type of recorded abuse was also requested for each case using the classifications within “No Secrets: Guidance on developing and implementing multi-agency policies and procedures to protect vulnerable adults from abuse” (Department of Health, 2000).(UK) | | Discriminatory abuse *4 items*: Racist, sexist, based on a person’s disability and other forms of harassment: slurs or similar treatment. |
| **Moore S 2020 UK [33]** | S | | Data gathering instrument | | NR |
| **Neuberg M et al. 2017 HRV[54]** | S | | Drennan J, Lafferty A, Treacy MP, Fealy G, Phelan A, Lyons I, Hall P. Older People in Residential Care Settings: Results from a National Survey of Staff-Resident Interactions and Conflicts. NCPOP: University College Dublin, 2012 Part 5: Staff interactions and conflicts with residents, including reports of neglect and abuse. | | NR |
| **Phillips LR & Ziminski C 2012 USA [40]** | ALL | | Review and copy all narrative reports for complaint investigations in public facility files. Types of neglect and neglect-related outcomes were identified from the complaint narratives using manifest content analysis  Categorised into Inappropriate care practices: Failure to meet scheduled and unscheduled needs, to provide safety and to notify appropriate persons in response to an emergency or accident, establish and maintain a written care plan, to correctly and safely administer appropriate medications, to provide appropriate nutrition and hydration and/or inappropriate admittance of a resident who does not meet ALF criteria | | NR |
| **Smith D et al. 2019 AUS (Women Only) [45]** | ALL | | Forensic medical reports (FMR) | | NR |
| **Smith DE et al. 2022 AUS [46]** | ALL | | Thirteen survey items were analysed: Perceived barriers to reporting Unwanted Sexual behaviour in residential aged care services. | | NR |
| **Teaster P B et al. 2015 USA [42] (Women Only)** | ALL | | Adult Protective Services (APS) and other regulatory entities from five states & used SASU | | NR |
| **Teaster P B et al. 2007, USA [41] (Men Only)** | ALL | | Adult Protective Services (APS) and other regulatory entities from five states & used SASU | | NR |

_NR = Not Reported; R = Resident; RT = Relatives; S = S_
